# Functional Diversity in Radiolabeled Nanoceramics and Related Biomaterials for the Multimodal Imaging of Tumors

**DOI:** 10.1021/acsbiomedchemau.3c00021

**Published:** 2023-08-08

**Authors:** David G. Calatayud, Marina Lledos, Federico Casarsa, Sofia I. Pascu

**Affiliations:** † Department of Inorganic Chemistry, Universidad Autónoma de Madrid, Madrid 28049, Spain; ‡ Department of Electroceramics, Instituto de Cerámica y Vidrio, Madrid 28049, Spain; § Department of Chemistry, University of Bath, Bath BA2 7AY, United Kingdom; ∥ Centre of Therapeutic Innovations, University of Bath, Bath BA2 7AY, United Kingdom

**Keywords:** multimodality imaging, iron oxide nanoparticles, nanoceramics, applied biomaterials, theranostics, targeted delivery, radio-nanomedicines, PET, SPECT, optical imaging

## Abstract

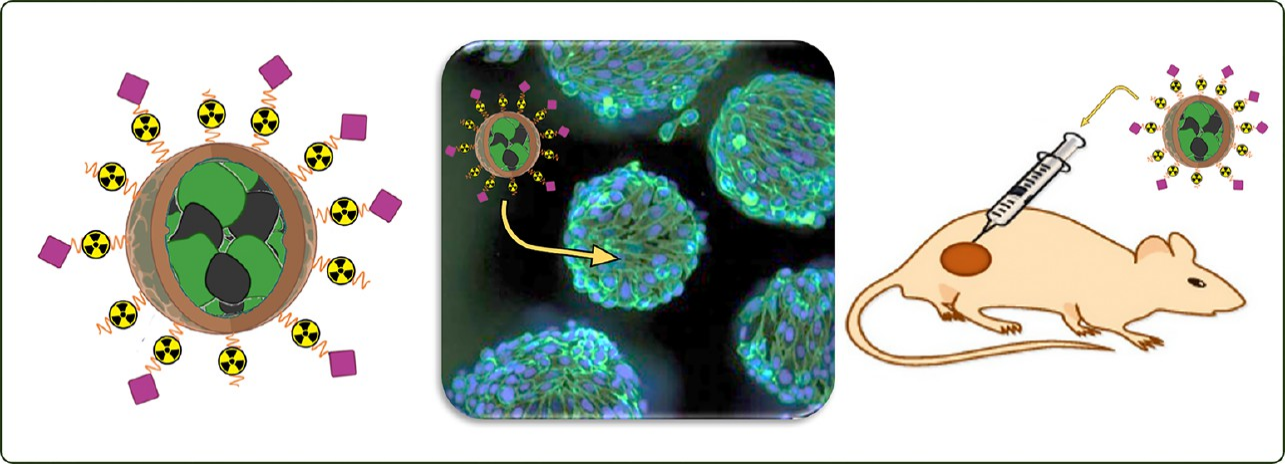

Nanotechnology advances have the potential to assist toward the earlier detection of diseases, giving increased accuracy for diagnosis and helping to personalize treatments, especially in the case of noncommunicative diseases (NCDs) such as cancer. The main advantage of nanoparticles, the scaffolds underpinning nanomedicine, is their potential to present multifunctionality: synthetic nanoplatforms for nanomedicines can be tailored to support a range of biomedical imaging modalities of relevance for clinical practice, such as, for example, optical imaging, computed tomography (CT), magnetic resonance imaging (MRI), single photon emission computed tomography (SPECT), and positron emission tomography (PET). A single nanoparticle has the potential to incorporate myriads of contrast agent units or imaging tracers, encapsulate, and/or be conjugated to different combinations of imaging tags, thus providing the means for multimodality diagnostic methods. These arrangements have been shown to provide significant improvements to the signal-to-noise ratios that may be obtained by molecular imaging techniques, for example, in PET diagnostic imaging with nanomaterials versus the cases when molecular species are involved as radiotracers. We surveyed some of the main discoveries in the simultaneous incorporation of nanoparticulate materials and imaging agents within highly kinetically stable radio-nanomaterials as potential tracers with (pre)clinical potential. Diversity in function and new developments toward synthesis, radiolabeling, and microscopy investigations are explored, and preclinical applications in molecular imaging are highlighted. The emphasis is on the biocompatible materials at the forefront of the main preclinical developments, e.g., nanoceramics and liposome-based constructs, which have driven the evolution of diagnostic radio-nanomedicines over the past decade.

## 1 Molecular Imaging Techniques Addressed by Nanoparticulate Tools

Molecular imaging is a general term describing a method for observing biological and physiological processes occurring within the living human body. This has been highlighted as one of the most inspiring and fast developing areas of science due to its “real life” applications,^1^ and it is an extension to the nuclear medicine field, which usually uses injected radiolabeled tracers in combination with technologies capable of obtaining an image. Clinically, the applications of molecular imaging depend on macroscopic-level transformations, be they of physical, physiological, or metabolic nature and often nonspecific, which indicate differences in the pathologically affected tissues compared to normal tissue. As such, medical imaging methods can give some detailed information relating to a particular disease state.^2^ Medical imaging techniques can be used as diagnostic tools in healthcare settings, and therefore, any advances in this area of science will be beneficial to the healthcare industry. Armed with greater knowledge of the biological processes occurring with disease progression, clinicians may be better positioned to determine an effective personalized treatment plan and achieve patient stratification.

**Figure 1:**
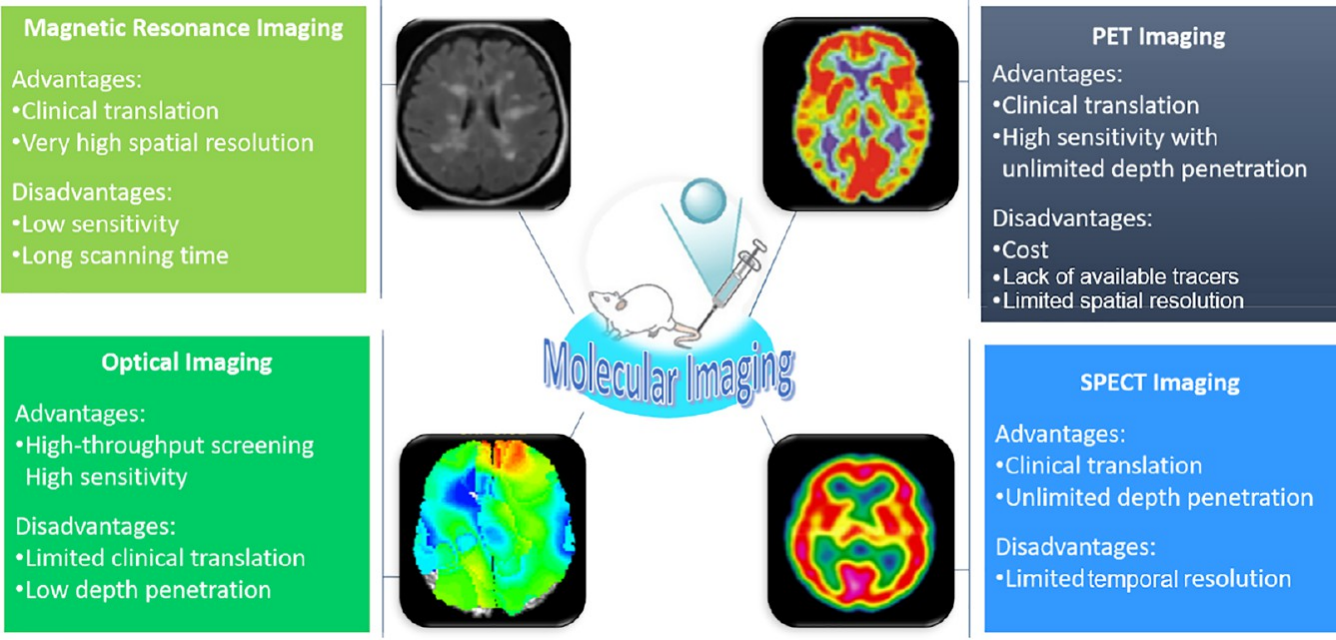
An overview of the main techniques used for molecular imaging in clinical practice, which will be the focus of this review. Adapted with permission from ref (3). Copyright 2008 Springer Nature.^3^

Molecular imaging techniques thus allow for the detailed and specific description and quantification of biological processes at a cellular level as well as *in vivo*, as depicted in Figure 1. Such clinically focused methods employ chemicals that are designed specifically to respond to the biological processes under study, act as tracers sensitive to intrinsic tissue features able to report upon, and thus help obtain “images” loaded with the crucial information necessary for detection/diagnosis and progression of a disease and evaluation of treatment.^3^

In the context of clinically relevant molecular imaging, and taking noncommunicable diseases, such as cancer, as a main focus of this review, there are currently several different diagnosis methods which are being mainly used in clinical applications for diagnosis:^4−9^

- Biochemistry-based testing: blood samples and/or other sample testing probes for the presence of biomarkers and/or targeting overexpressed molecules. These may be biomolecules such as sugars, fats, proteins, RNA, and DNA. While this is the first point of call in diagnosis, there is a lack of sensitivity and selectivity in the current testing reagents,^8^ especially for cancers such as prostate cancer, and screening using these methods remains a detection goal.
- Biopsy: this remains the most common way to diagnose cancer; however it is deemed highly invasive as the procedure consists of the collection of a tissue sample from the site of interest for a subsequent biological/histological examination, e.g., involving the optical imaging of the tissue morphology as well as the determination of gene status.
- Endoscopy: depending on the nature of the cancer and its symptomatic presentation, this method is generally widely available yet applied at the middle or late stages of cancer diagnosis. Its diagnostic and prognosis relevance is in combination with approaches (a) and (b) and coupled with complementary imaging tools (such as molecular imaging, (d)). As such, it is widely applied in clinical practice to confirm a cancer diagnosis and/or in conjunction with biopsy to collect a tissue sample for further investigations.^10^
- Medical imaging, or molecular imaging, methods are less widely available compared to (a)–(c) in practical terms; however they have been deemed beneficial to employ in the cases that the location of a tumor in a specific site is difficult, such in the case of a difficult to access tumor (e.g., prostate, esophageal cancers). Molecular imaging has the capacity to speed up the diagnosis combined with blood sample testing to enable the location of a tumor or early cancer detection.^10^

Aspects of medical imaging techniques and the development of relevant chemistry-focused tools constitute one of the main research frontiers that we were interested in, and contributed through doctoral thesis programs and thematic reviews over the years: the most recent advances in this field, with an overview of the work published in the past decade, are the focus of this review.^9,11−21^

Molecular imaging modalities, which we intend to touch upon hereby from the perspective of nanochemistry tools, and recent developments include optical fluorescence, magnetic resonance imaging (MRI), single photon emission computed tomography (SPECT), and positron emission tomography (PET).^22^ Although a wide range of new molecular imaging techniques have emerged and are of relevance in preclinical studies, for example, photoacoustic imaging, and which we will consider elsewhere.

As stated above, medical imaging techniques most commonly used in cancer detection, diagnosis and monitoring the therapeutic effects include X-ray computed tomography (through CT scans), MRI, SPECT, and PET.^23−26^ These techniques are generally reliant on the use of a source of energy (e.g., X-ray, magnetic fields, gamma or positron decays) to create comprehensive images of a living subject, with the purpose of locating a tumor mass. As such, they can be used to detect all types of cancer. However, there are advantages and disadvantages behind their applicability and accessibility in all of these methods.

Recent advances in the availability of imaging probes and highly specific probes have meant that molecular imaging has developed into an area of research of high interest. There are many advantages in combining modalities in the context of molecular imaging, as it allows one to combine the advantages of each technique and save or minimize their disadvantages. For example, PET is a quantitative technique, whereby the resulting images are not subjective or qualitative in nature and rather represent the data collected and reconstructed/interpreted through meaningful numerical measurements of a biological process. This feature also allows for more thorough determination of the biological processes occurring in a living subject compared to *in vitro* and cell culture techniques.^27^ Nuclear imaging can provide information noninvasively and the high sensitivity means that nano- or even picomolar concentrations of the imaging agents used as tracer can be used to achieve acceptable signal-to-noise ratios, and hence increasing the accuracy of the diagnosis of a disease, of particular relevance to cancer detection. The small concentrations of imaging agent used also means that the risk of adverse pharmacological effects may be reduced.^2^ In the particular case of nanomaterials, the administered concentrations are higher, because contrary to small molecules, the labeled species cannot be easily separated from the nonlabeled species.

**Figure 2:**
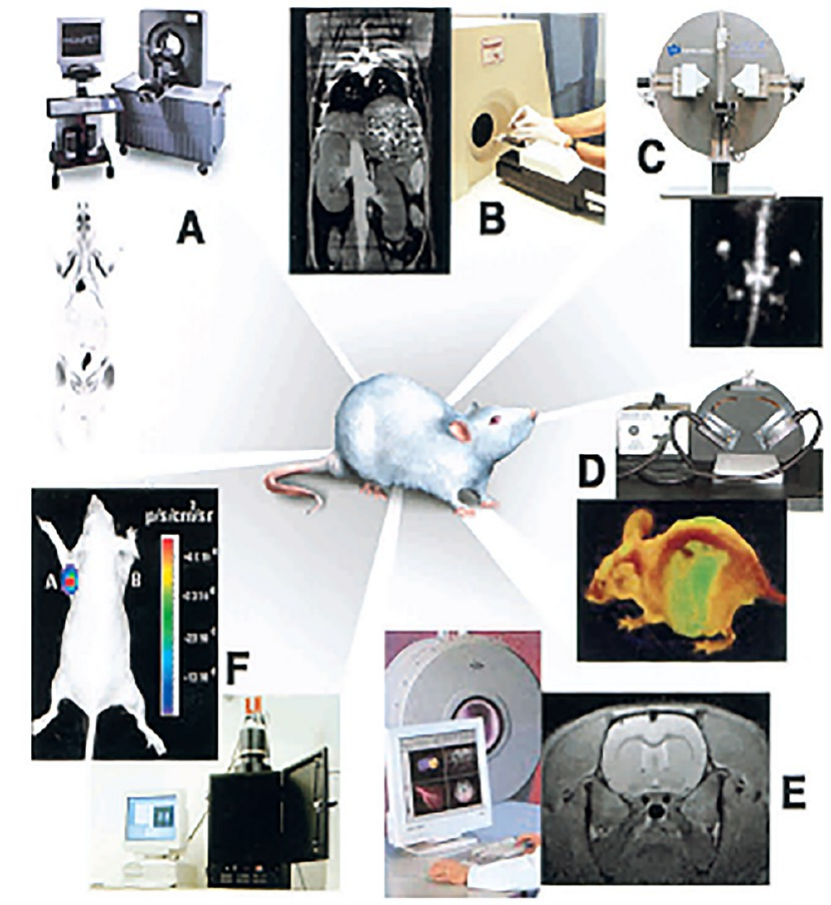
Early example focused on preclinical investigations indicative of a feasible way to combine molecular imaging modalities. Reproduced with permission under a Creative Commons CC-BY License from ref (27). Copyright 2003 CSH press.^27^

Figure 2 reveals an overview of these imaging modalities in the interlinked perspective of Massoud and Gambhir.^27^ As highlighted by these authors, Figure 2(A) shows the image taken by the whole-body microPET and representing the coronal image of a rat injected with a radionuclide that localized in various tissues and also accumulated in the bladder. The related Figure 2(B) shows a microCT coronal image of a mouse abdomen after injection of intravenous iodinated contrast agent, whereas Figure 2(C) shows a microSPECT coronal image of a mouse abdomen and pelvis regions after injection of a radionuclide that accumulates in the bones. Coupled to these, Figure 2(D) shows an optical reflectance fluorescence image of a mouse presenting fluorescence emitted from the liver, abdomen, spine, and brain areas, which the authors assigned to the presence of a specific type of tumor cells. Figure 2(E) shows a microMRI coronal T2-weighted image of a mouse brain and this is complemented by Figure 2(F), which shows an optical bioluminescence image of a mouse.^27^ These images highlighted by the authors represent one of the early overview where it was possible to link various modalities in preclinical approaches.

A very summary overview of medical imaging approaches, taken from the perspective of the interface between physical and life sciences is highlighted below to set the scene of this review at the interface between disciplines.^9,11−21^

### 1.1 MRI as a Diagnostic Imaging Modality: Basic Considerations

*Magnetic resonance imaging (MRI)* depends on the basic physical principles underlining the nuclear magnetic resonance (NMR) which is a spectroscopic technique that allows chemical and physical information to be obtained regarding the structure of a molecule, and it is widely used in physical sciences research across all fields. While NMR has the capability to provide chemical information from a whole sample, rather than provide detail information about the internal structure of a sample, and led to the award of the Nobel prize to Peter Mansfield and Paul Lauterbur in 2003.^28^

MRI delivers images based on spatial variations in the phase and frequency of radiofrequencies (RF) that are being absorbed and emitted by the imaged object and is a primary diagnostic tool in clinical practice. This is because living organisms (e.g., the human body) tissues comprise primarily water and fat molecules, and chemically these species are rich in hydrogen: this element therefore constitutes up to 63% of the human body, by mass.^29^ Each proton possesses its own magnetic moment and is randomly oriented in the absence of magnetic *stimuli*, yet by applying a strong, external magnetic field, the protons in turn assume a nonrandom alignment. This results in a measurable magnetic moment in the direction of the external magnetic field. Furthermore, after the application of RF pulses, images emerge and can be reconstructed: these images derive from the discrepancies in signal from protons in different types of tissue, and several scanning techniques have been developed to enhance the MRI effectiveness. The main advantage of MRI over other medical imaging techniques is its very high spatial resolution, which is assigned to the superior soft tissue contrast resolution and multiplanar imaging capabilities.^30^ Conveniently, MRI scanning in patients does not require the use of ionizing radiation, and this technique is now widely used in clinical settings for medical diagnosis, staging of disease, and follow-up post-treatment, without the drawbacks of potential exposure to harmful radiation. However, the main drawback of MRI for clinical applications is its very low sensitivity, ca. 10^–3^ to 10^–5^ mol/L, which has been shown to be well below that of nuclear imaging techniques such as PET and SPECT.^31^

The enhanced sensitivity makes it possible to take advantage of the great spatial and temporal resolution of the MRI imaging modality, thus allowing a detailed picture of the biological microenvironment to be acquired at the cellular and molecular level. The use of contrast agents in MRI allows enhancement of the signal for certain types of tissues, organs, or molecules by altering the longitudinal and transverse relaxation time (T1 and T2) of H_2_O protons within these systems. This is traditionally achieved by using paramagnetic metal ions such as gadolinium (Gd^3+^)^32^ and agents such as the ferrocene-conjugated complex Gd-DTPA, developed by Kim and co-workers,^33^ further demonstrated high relaxivity as well enhanced thermal and kinetic stability in the target tissue.

The development of molecular imaging techniques currently progresses toward identifying molecular abnormalities that form the bases of a disease, rather than observing the consequences of a disease as it progresses. If this can be achieved, then molecular imaging will allow earlier detection and identification of a disease.^9,19,34^ One way in which this can be reached is by combining two or more molecular imaging techniques, as no single imaging technique currently delivers full understanding of local tissue environments.

The need to adopt synergetic approaches has opened the way forward for nanomedicine development. Nanoparticles hybrids with a promise to increase the technical advantage of MRI have been recently developed whereby nanomaterials combining fluorescence and magnetic resonance imaging were found to tie the high sensitivity of fluorescence and the high spatial resolution of MRI.^35^ The area remains a vivid subject of investigation from the perspective of physical and life sciences as well as preclinical/clinical applications as well as subject to topical reviews especially in the context of materials development,^9^ and further details in the context of multimodality will be explored below.

### 1.2 Nuclear Medical Imaging: PET and SPECT

PET imaging has a key role in molecular imaging as it provides much more than just the structural information that can be obtained from MRI and CT. In addition, the combination of nuclear imaging techniques, such as positron emission tomography PET and MRI, achieves the high soft tissue contrast of MRI and the functional information on PET, which means that the final data are detailed information on anatomy and function, and the area has been the subject of reviews.^9,34^

PET images are generated by high-energy γ-rays that are emitted by radioisotopes. Radioisotopes emit positrons from within the nucleus, and when a positron collides with an electron, two γ-rays are produced, and the positron and electron are annihilated. The biologically active molecule along with the radioisotope is called a tracer. These tracers can be designed to target specific cells and accumulate there, and then images can be taken of the area to determine the biological processes occurring in that specific tissue. Radioisotopes provide a route for studying human anatomy and physiology. By measuring physiological functions and biochemical parameters that are known to be involved in human disease, such as enzymatic reaction rates or cell surface receptor densities, information can be gained, being invaluable to the treatment of a disease.^36^ These isotopes may be delivered as biologically active molecules, which are introduced into a subject. In clinical practice, the most commonly used positron-emitting radioisotopes^37^ include, e.g.m ^15^O (*t*_1/2_ = 122.266 s), ^13^N (*t*_1/2_ = 9.97 min), ^11^C (*t*_1/2_ = 20 min), ^18^F (*t*_1/2_ = 109.771 min), ^64^Cu (*t*_1/2_ = 12.701 h), ^62^Cu (*t*_1/2_ = 9.67 min), ^124^I (*t*_1/2_ = 4.2 days), and ^68^Ga (*t*_1/2_ = 68 min), and recent preclinical interest expanded upon ^52^Mn (*t*_1/2_ = 5.591 days) and ^89^Zr (*t*_1/2_ = 3.3 days) labeling for antibodies and small molecules as well as in nanotechnology-driven developments. This latter aspect will be outlined in more detail here.

To obtain the optimum patient outcome from PET imaging, the choice of the radiotracer is crucial. In recent years, many radiolabeled compounds have been synthesized in order to improve their localization and the detection of cancers.^38−41^ The most commonly used small-molecular radiotracer for PET imaging is 2-[^18^F]fluoro-2-deoxy-d-glucose ([^18^F]FDG] or ^18^F-FDG). This radiolabeled analogue of glucose was developed as a tracer selective for high-glucose-utilizing cells such as brain, kidney, and cancer cells. In clinical imaging, [^18^F]FDG may be used for the detection of hidden metastatic lesions in patients with biochemical recurrence (a state characterized by an increasing level of prostate-specific antigen) and the evaluation of the treatment response in advanced prostate cancer.^39^ Interestingly, FDG was originally developed as DG (2-deoxy-d-[^14^C]glucose), and it was designed to prevent accelerated cell growth found in cancerous tumors. However, DG was found to have adverse effects in the brain and hence never made it into the pharmaceutical market. It was later adapted to FDG, which was designed to specifically image living subjects noninvasively with PET.^37^ For suitable images to be taken, it usually requires several hundred million cells in close proximity to have taken up the tracer. It does however provide images with high sensitivity normally between 10^–11^ and 10^–12^ mol/L, which is much greater than that routinely provided by MRI or CT and with relatively high resolution. The relatively short half-life of the cyclotron-available isotopes brings advantages and disadvantages; ^18^F has a half-life of ca. 110 min, which means there is a short window of opportunity for the tracer to be synthesized, transported, and introduced into a living subject, to reach the target tissues and accumulate to a concentration that allows the images to be taken. However, the short half-life is also advantageous in that it reduces the risk of accumulation of the radioisotope which could lead to adverse pharmacological effects or toxicity, a key criterion when developing a material that can be used in humans.^37^ PET tracers incorporating ^64^Cu (a copper isotope which undergoes β^–^ electron capture and positron decay leading to β^–^ emissions and Auger electrons) may be utilized for therapy and simultaneously considered as a basis for “true theranostics” because of their usefulness in tomographic imaging. ^64^Cu also has a half-life of 12.7 h giving the radioisotope a large enough window of opportunity for the radiopharmaceutical synthesis especially as the ^64^Cu labeled diacetyl-bis(*N*-methylthiosemicarbazone) (^64^Cu-ATSM)^42^ and delivery to patients.^43^ As pointed out in a seminal review on nanoparticles labeled with PET emitting radionuclides by Liu and Welch in 2012,^44^ multimodality methods underlined by the employment of multifuctional nanoparticles for cardiovascular imaging, lung diagnosis, and tumor theranostics are holding significant promise. The 100 nm diameter of such nanoparticles was considered ideal to ensure prolonged blood circulation and a low rate of mononuclear phagocyte system (MPS) uptake.

**Figure 3:**
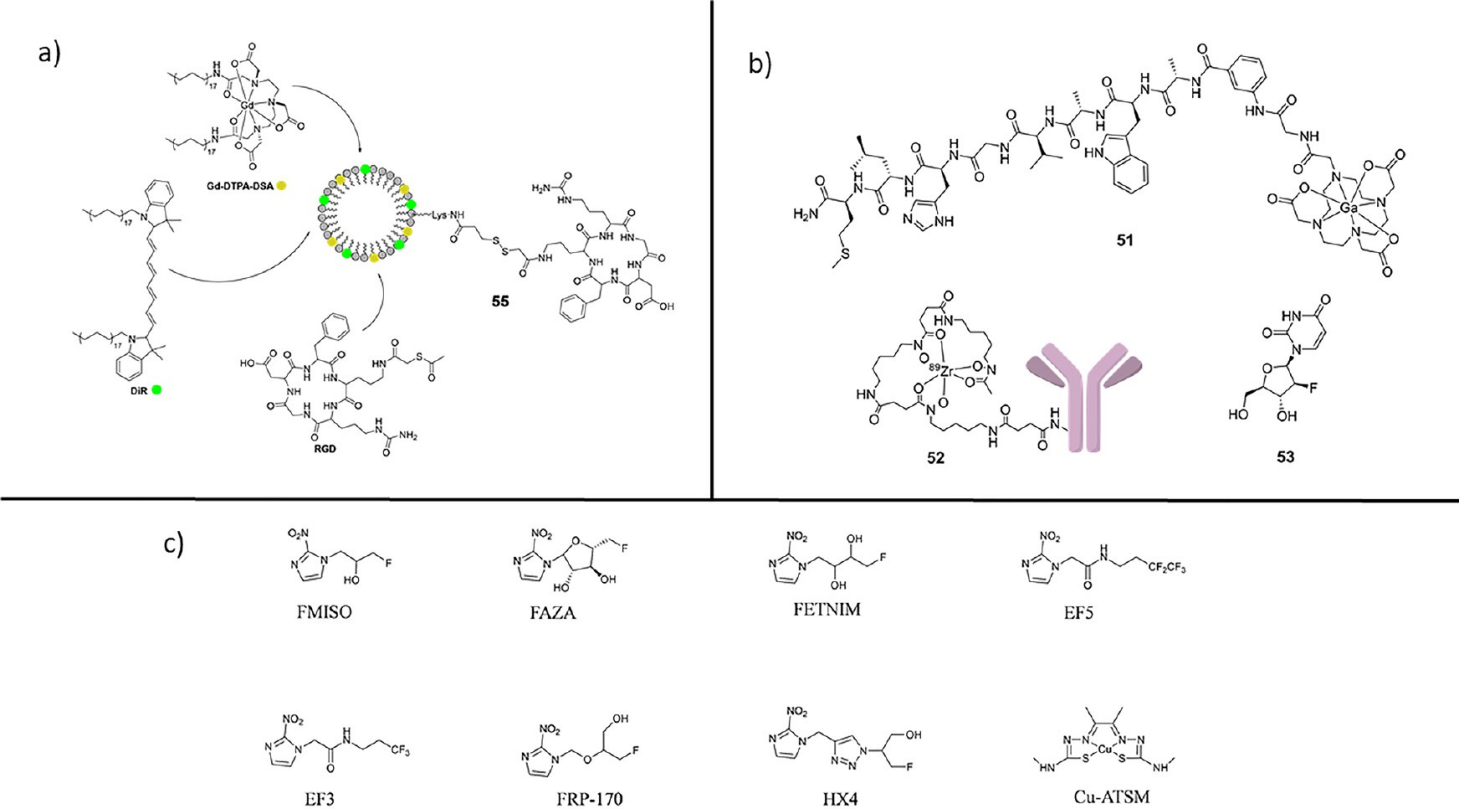
(a) Structural representations of the small-molecular tags ^68^Ga-DOTA-CHCO-Gly-4-aminobenzyl bombesin (**1**), ^89^Zr-5A10 monoclonal antibody (**2**), and 1-(2′-deoxy-2′-fluoro-*b*-d-arabinofuranosyl) thymidine (**3**). (b) Structural representation of a multifunctionalized nanoparticle and its constituents: Gadolinium diethylenetriaminepentaacetate-di(stearylamide) (Gd-DTPA-DSA, yellow dot) as MRI contrast agent, 1,1′-dioctadecyl-3,3,3′,3′-tetramethylindotricarbocyanine iodide (DiR, green dot) as NIR dye, and the cyclic RGD-containing pentapeptide (c(RGDf(S-acetylthioacetyl) K) (RGD) as specific targeting agent. (c) Structural representations of clinically relevant small molecular PET radiotracers. ^18^F-fluoromisonidazole (FMISO), ^18^F-fluoroazomycin-arabinofuranoside (FAZA), ^18^F-fluoroerythronitroimidazole (FETNIM), [^18^F]-2-(2-Nitro-1*H*-imidazol-1-yl)-*N*-(2,2,3,3,3-pentafluoropropyl) acetamide (EF5), EF3, RP-170 (1-(2-1-(1*H*-methyl) ethoxy)-methyl-2-nitroimidazole) (FRP-170), 3-[^18^F]-2-(4-((2-nitro-1*H*-imidazol-1-yl) methyl)-1*H*-1,2,3,-triazol-1-yl)-propan-1-ol (HX4), copper-labeled diacetyl-bis(*N*-methylthiosemicarbazone) (Cu-ATSM).

In terms of design elements of relevance for radio-nanoparticles as radio-nanomedicines, an essential consideration needs to be given to the components assembled and also to the comparison with the small molecular species that already perform this function as either diagnostics or therapeutics and are already adopted in clinics. To add this perspective, in this review, we also include some of the simpler molecular species that have already been adopted in clinical practice for PET diagnostics. This is because the ideal NP size places these at the frontier between the materials (where macroscopic level properties prevail) and small molecules (Figure 3a); yet they still exhibit molecular-level detail on the surface.^45^ As such, they can be designed and engineered in ways in which the characteristics of both macroscopic materials and molecular systems are represented. Their size is comparable to large biological molecules (monoclonal antibodies MABs, DNA/RNA fragments), and at the same time nanoparticles can interact with various biomolecules situated on the surface of cells, inside the cells, and/or within tissues and organs, leading to significantly differing potential for diagnosis and treatment efficacy when compared to small molecular drugs and/or bulk, macroscopic-level materials.^44,45^

In terms of nanoparticulate analogues for PET imaging, a whole range of inorganic nanoparticles has been developed and reported starting from about a decade ago, in particular, those incorporating ^18^F.^46^ Most recently emerging were radiotracers designed to target surface receptors that are generally upregulated in cancer cells, and these constituted attractive targets for therapy and diagnosis. For example, prostate-specific membrane antigen (PSMA) based radiotracers have been developed, including peptidomimetic PSMA inhibitors and radiolabeled antibodies.^47^ Promising derivatives include the bombesin-based ligand ^68^Ga-DOTA-CHCO-Gly-4-aminobenzyl-Gln-Trp-Ala-Val-Gly-His-Leu-Met-NH_2_ (Figure 3) that binds to the overexpressed gastrin-releasing peptide (GRP) receptor, the ^89^Zr-5A10 monoclonal antibody that targets free prostate-specific antigen (PSA), and the 1-(2′-deoxy-2′-fluoro-*b*-d-arabinofuranosyl) thymidine, specific for thymidine kinase, which was developed for assessing cellular proliferation and as a cellular stress marker.^48^

There are three molecular-imaging relevant zirconium isotopes that can be produced using different nuclear reactions, and with particle energies between 5 and 85 MeV (Table 2).^49^ Among these, ^89^Zr is the most promising one for investigating new immunoPET agents to use in *in vivo* imaging of cancerous tumors and to guide and plan radioimmunotherapy. New clinical and preclinical studies emerged with ^89^Zr-labeled antibodies: this long-lived radioisotope with half-life of 78.4 h allows PET imaging after several days, on a time scale that is comparable to the time necessary to realize the optimal tumor-to-background ratios for intact proteins in circulation in living systems, such as, for example, monoclonal antibodies.^50^ An early example of ^89^Zr-labeled cross-linked dextran nanoparticle showed primary localization in lymph node as well as intense tumor uptake (at 20 ± 5%ID/g), which was higher than the uptake in other mononuclear phagocyte system (MPS).^51^

Other interesting engineered ^89^Zr NPs which indicated relevance to dual modality approaches have also been reported. The intrinsic labeling with ^89^Zr of a PEG-ylated Gd_2_O_2_S:Eu nanophosphor formed the radio-nanohybrid denoted [^89^Zr]Gd_2_O_2_S:Eu@PEG, which showed promising *in vivo* PET/radioluminescence lymph node mapping *in vivo*.^52^

**Figure 4:**
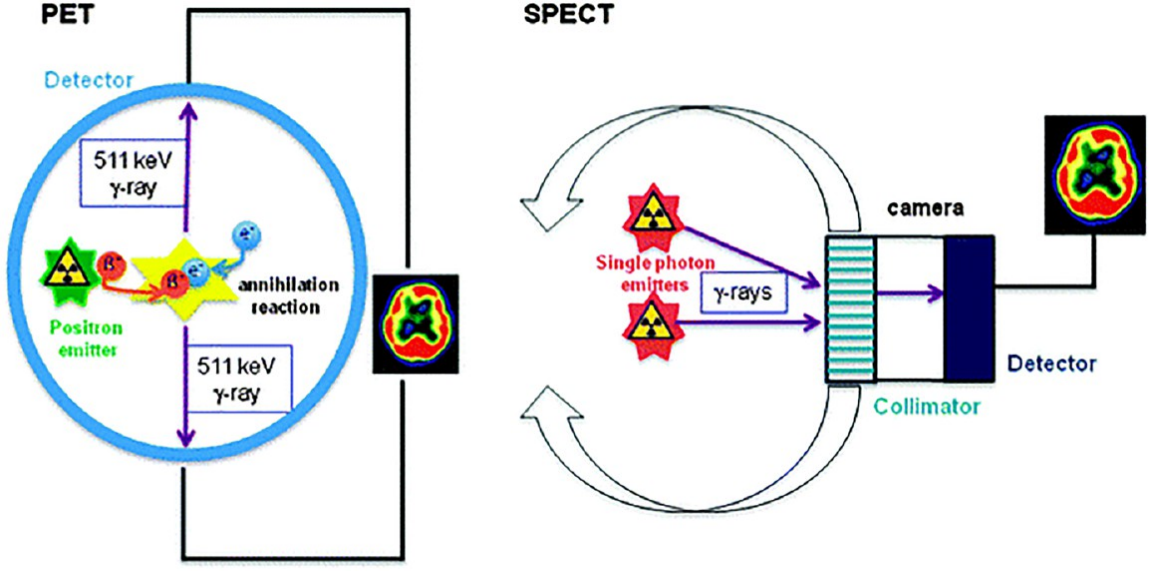
Schematic representations for PET and SPECT applications. In PET, the emitted positron undergoes an annihilation process with an electron, thus giving rise two γ-rays situated at 180° from each other. Their emergence is detected, and a 3D image of the tracer concentration is obtained by software reconstruction.

Further examples of multimodality approaches for radio-NPs will be highlighted in the dedicated section below. Similarly, to PET, nuclear imaging using SPECT employs a radioisotope that emits one or more γ-rays of characteristic energies, which are then directly measured by an instrument (Figure 4).^53^ The generally accepted advantages of this technique, compared to PET, are lower cost, thanks to the long half-lives of the radioisotopes used, as ^99m^Tc *t*_1/2_ = 6 h (with a widespread use as it can be produced in generator), and the possibility of using different isotopes in the same study (Table 1). In contrast to PET, SPECT imaging suffers from lower temporal resolution, and the use of heavier isotopes may alter the biochemical properties of the labeled compounds; as such, PET is considered as the more robust technique for the imaging of molecular events *in vivo* (Table 3). SPECT diagnosis is however more clinically available: it can also be used with tracers for imaging living subjects with a different type of cameras (“gamma-cameras”), which do not require the production of two coincident γ-rays.

**Table 1:** Side-by-Side Comparison of the Most Common Radioisotopes Used in PET and SPECT in Clinical Settings^a^

Isotope	Half-life	β^+^ Energy (MeV)	γ Energy (MeV)
^11^C	20.4 m	0.385 (99.8%)	
^13^N	9.97 m	0.492 (99.8%)	
^15^O	122 s	0.735 (99.9%)	
^18^F	109.7 m	0.250 (100%)	
^38^K	7.64 m	1.216 (99.3%)	2.167 (99.8%)
^62^Cu	9.67 m	1.315 (97.6%)	
^64^Cu	12.7 h	0.278 (17.9%)	
^68^Ga	68.1 min	0.836 (8.79%)	1.077 (3.0%)
		0.352 (1.12%)	
^82^Rb	75 s	1.523 (83.3%)	0.776 (13.4%)
		1.157 (10.2%)	
^124^I	4.18 d	0.686 (11.3%)	1.691 (10.4%), 7.228 (10.0%), 1.509 (3.0%), 1.376 (1.7%), 1.325 (1.43%)
		0.974 (11.3%)	

a The average energy of the positron (β+) is given along with the percentage of decays in which the β+ is emitted. The energy of γ-rays that occur in more than 1% of decays is given along with the percentage of decays in which γ-rays are emitted.

**Table 2:** Properties of Selected Zirconium Isotopes^a^

Isotope	*t*_1/2_	*I*_*γ*_	*E*_γ_	*I*_ec_	*I*_β+_	*E*_max__(β+)_	*E*_ave(β+)_
^86^Zr	16.5 h	100%	241 keV				
^88^Zr	83.4 d	100%	390 keV				
^89^Zr	78.4 h	100%	909 keV	76.6%	22.3%	897 keV	397 keV

a *t*_1/2_ is the half-life of the radioisotopes; *I*_γ_ and *E*_γ_ refer to the intensity and energy of the γ emission, respectively; IEC denotes the intensity of the electron capture decay; *I*_β+_ is the intensity of the positron emission decay, and *E*_max(β+)_ and *E*_ave(β+)_ designate the maximum and average energies of the decay by positron emission, respectively.^49^.

**Table 3:** General Overview and a Very Basic Comparison of PET and SPECT Modalities from Their Clinical Availability Perspective^a^

	SPECT	PET
Type of radioisotope used	Photon emitter	Positron emitter
Average half-lives of commonly used radioisotopes	Hours to days	Seconds to minutes^a^
Examples of isotopes	^99m^Tc, ^201^Tl, ^131^I, ^111^In, ^123^I, ^133^Xe	^18^F, ^11^C, ^13^N, ^15^O, ^68^Ga
Spatial resolution	*x*	3*x*
Contrast resolution	*x*	2*x*
Signal noise:ratio	*x*	2*x*
Variety of ligands	Under development	Higher diversity
Availability	Widely available	Highly restricted
Sensitivity	High	Very high

a Note that in preclinical application, long-lived radioisotopes such as ^64^Cu (*t*_1/2_ = 12.7 h), ^89^Zr (*t*_1/2_ = 3.27 days), and ^52^ Mn (*t*_1/2_ = 5.59 days) are becoming more widely available.

In clinical practice, SPECT has been used to detect bone metastases in patients with advanced prostate cancer: for example, radiolabeled phosphonates such as the ^99m^Tc-diphosphonate have been developed and used in diagnosis.^54,55^ Commonly investigated γ-emitting isotopes, such as ^99m^Tc, ^111^In, ^123^In, ^131^I, and ^67^Ga, are suitable for imaging living subjects using single photon emission computed tomography (SPECT). ^99m^Tc is the most used isotope in SPECT: it has a half-life of 6 h which is convenient for pharmaceutical preparation and formulation and allows for the *in vivo* accumulation at the target tissue. Although the concentration of tracer needs to be sufficient to allow for imaging, it is important to note that these tracers are given in nanomolar quantities or less which significantly reduces the risk of radiotoxicity.

SPECT imaging has a higher spatial resolution than PET, because one does not have the positron range and can use pinhole collimators (which narrows a beam of particles: either to cause the directions of motion to become more aligned in a specific direction or to cause the spatial cross section of the beam to become smaller). In SPECT, a compromise is always required between spatial resolution, field of view, and sensitivity, and temporal resolution is a stronger limitation of SPECT.^56,57^ The synthetic strategies for a range of ^99m^Tc radiolabeling strategies for inorganic and organic nanoparticles and their application to preclinical imaging studies has been reviewed from the perspective of the comparison between ^98m^Tc-radiolabeled small molecules such as chelators, corresponding biomolecules (e.g., ^99m^Tc(CO)_3_-tagged anti-CD20 IgG antibody), and ^99m^Tc(CO)_3_-linked to Au-Fe_3_O_4_ nanoparticles.^58^

### 1.3 Optical Imaging and Theranostics with Organic Fluorescent Tags

Molecular imaging (MI) is a growing biomedical research discipline that enables the visualization, characterization, and quantification of biologic processes taking place at the cellular and subcellular levels within intact living subjects, including patients. Molecular imaging originated in the field of nuclear medicine and has now developed to include an array of different strategies to produce imaging signals. Whereas nuclear medicine uses radiolabeled molecules (tracers) that produce signals from radioactive decay only, MI uses these and other molecules to image via sound (ultrasound), magnetism (MRI or magnetic resonance imaging), or light (optical techniques of bioluminescence and fluorescence), as well as other emerging techniques, e.g., photoacoustic imaging, Raman spectroscopy, and amide proton transfer imaging.

Molecular imaging techniques and blood sample tests speed up the diagnosis of cancer in an early stage by locating the cancer: a classical method utilizes organic fluorophores or quantum dots (QDs) as staining reagents for biological assays. More recently, optical fluorescence imaging was considered for its potential to locate and image tumors *in vivo* with near-infrared (NIR) imaging investigations by using advanced endoscopy: this is of relevance for the diagnosis of difficult-to-access cancers such as colorectal cancer or cancer of the esophagus. In this review, we will focus on the complementarity that the optical imaging techniques may offer to other imaging methods, rather than on advancements in optical imaging *per se*; however, the relevance of this technique is inevitably closely intertwined within the multimodality aspects, highlighted below. Coupled to this, optical imaging and photodynamic therapies were closely interlinked in theranostics developments: the process of photochemical reactions generates singlet oxygen from ^3^O_2_ which is responsible for tissue damage in the regions.^59,60^ The organic dyes BOPIDY is a commonly used category of imaging and photodynamic therapeutics (PDT) agents, which depresses fluorescence and enhances singlet to triplet intersystem crossing; however, it was also widely used in conjunction with radiolabeled probes.

In comparison with other fluorescent dyes, boron–fluorine dyes have a large molar absorptive coefficient (>8 × 10^4^ cm^–1^ M^–1^), high chemical stability and photostability, and are amenable to facile structure modifications.^61^ Moreover, BODIPY as a fluorescent dye shows small Stokes shift, good photochemical stability, high fluorescence quantum yields, and sharp excitation–emission peaks. Their unique photophysical properties make them suitable to use for bioimaging and to be applied as a sensor.

Some studies emphasized the combination between BODIPY analogues and coated iron oxide nanoparticles (IONPs) with carboxylic acid at the surface area, which is applicable to humans with cancer cells. The cytotoxic activity of the BODIPY conjugated with iron oxide nanoparticles was tried in healthy human cells human umbilical vein endothelial cell line (HUVEC), in A549 and Ishikawa cells by standard MTT (3-(4,5-dimethythiazol-2-yl)-2,5-diphenyl tetrazolium bromide) assay.^62^

The main physicochemical characteristics of BODIPY include its high efficiency, environment insensitivity, resistance to photobleaching, and higher light–dark toxicity than other commonly used PD therapeutics. BODIPY is considered a more desirable fluorescent probe rather than fluorescein and rhodamine, as it has high photostability, neutral total charge, and high fluorescence and emission spectra. For example, in cellular imaging techniques, rhodamine was shown that sometimes is absorbed by nonspecific proteins and lipids which causes imaging and localization challenges.^60,63−65^ Advantages of PDT with optical probes such as BODIPY led to applications in targeting sites which are difficult to access by surgery, do not produce immunosuppression, and can be used in combination with chemotherapy and radiotherapy with a synergistic effect.^59,66^

Optical imaging (OI) is a noninvasive imaging technique of relevance to diagnostics and in combination with other modalities (under the wider multimodality umbrella) that allows the visualization and imaging of the biodistribution of fluorescent molecules in living organisms. OI makes use of an external excitation light source (usually a laser) that excites a selected fluorescent probe that emits light at a longer wavelength of lower energy. This type of imaging is particularly useful for monitoring the targeted accumulation of fluorophore-labeled drugs thanks to its high sensitivity and resolution, as well as the possibility to perform multimodal imaging by coupling OI with other imaging techniques, such as computed tomography and PET. Lack of deep tissue penetration, autofluorescence, and diffusive scattering phenomena and the lack of anatomical information are the main limitations of OI; however, these are now being addressed by expansion of the imaging window into the NIR, with the design and delivery of new organic fluorophors based on the cypate family.^67^

While the majority of optical imaging (OI) applications are in preclinical research (using techniques such as confocal microscopy), it is possible to exploit OI for clinical purposes.^68^ A report by van Dam et al. highlighted a methodology to exploit the near-infrared fluorescent dye derivative named Folate-FITC, which consists of fluorescein coupled with a targeting molecule specific for the folate receptor-α (overexpressed in ovarian cancer cells). The folate-FITC in an intravenously injected formulation enhances the fluorescence imaging over the time course from 2 to 8 h after injection. This could be used to help surgeons detect and remove malignant lesions while keeping as much healthy tissue as possible.^69^ Overall, the OI displays excellent sensitivity, and it is particularly suitable for noninvasive imaging of drug localization in superficial tumors. Due to the excessive light scattering effect of deep tissues, such a technique is usually restricted to superficial tissues where the light can easily penetrate. Table 4 and associated references included below provide an overview of the theranostic nanomaterials that are currently most applicable clinically (or are under trials) as well as those in preclinical studies for imaging and which we surveyed hereby.

**Table 4:** An Overview of the Nanorelated Materials Developed Used in Imaging Applications and Corresponding References

NP type (abbreviated name from state-of-the-art)	Detection mode	*In vitro*/*in vivo* applications	Reference
PMAO-(β-NaY_0.78_Yb_0.2_Er_0.02_F_4_)-Bs:Bn-scFc_4_D_5_	Fluorescence/optical imaging	Detecting early stage breast cancer	(164)
Cy5.5-substrate/AuNP	Fluorescence/optical imaging	Detecting protease activity	(165)
Cy5.5-DEVD-DOPAK/AuNP	Fluorescence/optical imaging	Testing caspase-3 to identify apoptosis activity in cells	(166)
PLNP(Zn_1.1_Ga_1.8_Ge_0.1_O_4_:Cr^3+^)-CuS-RGD	Fluorescence/optical imaging	Detecting tumor and guiding therapy	(167)
DNAzymes(Zn-Enz)/AuNP-FAM/BHQ-1, DNAzymes(Cu-Enz)/AuNP-Cy5/BHQ-2	Fluorescence/optical imaging	Tracking ion of Zn and Cu in alive cell	(168)
CNP(Mtx-Asp-FITC)	Fluorescence/optical imaging	Monitoring therapeutic drug delivery	(169)
Cy7.5-INCeRT	Fluorescence/optical imaging	Monitoring drug diffusion	(170)
QD710-Cy7-PEGylated lipids	Fluorescence/optical imaging	Monitoring NP accumulation and dissociation kinetics in tumor	(171)
QD710-Dendron/RGD (InP/ZnS core/shell QDs)	Fluorescence/optical imaging	Targeted imaging tumor cells	(172)
Quantum Dots	Fluorescence/optical imaging	Preclinical imaging	(173)
Cationic oligofluorene substrated POSS	Ethidium bromide test	Imaging double-stranded DNA	(174)
Perylenediimide-containing polysiloxane core and silica shell	Perylenediimide toxicity	Detecting nanotoxicity in living cells	(175)
AB3-UCNP(NaYF_4_:Yb/Tm/Er)-RB/KE108	Up-Converting NP celular imaging	Monitoring cellular uptake of nanoparticles and combined with therapy	(176)
Au@IR-pHPMA	IR	Detecting lymph node	(177)
Gadolinium nanostructure polymers, liposomes, inorganic nanoparticles	MRI imaging	Preclinical	(178)
Superparamagnetic iron-oxide nanoparticles coated with dextran	MRI imaging	Clinical use; FDA Approved (Feridex/Endorem)	(179)
Bismuth sulfide (Bi_2_S_3_) nanoparticles	CT scan	Preclinical	(180)
Iodinated liposomal carriers, inorganic nanostructures	CT scan	Preclinical	(181)
Gold particles	CT scan	Preclinical	(182)
Alpha(nu) beta(3)-Gd (paramagnetic particle)	MRI imaging	Imaging angiogenesis	(183)
Liposomal gadolinium	MRI imaging	Imaging placenta as blood-pool contrast	(184)
Her2/neu-Oleosin-30G (Micelles)	MRI imaging	Imaging target cells	(185)
G4.5-Gd_2_O_3_-PEG	MRI imaging	New T1/T2MRI contrast agent	(186)
SPIO	MRI imaging	Tracking GFP gene marker	(187)
rHDL-Gd	MRI imaging	Imaging and characterizing atherosclerotic plaques	(188)
RBC encapsulated iron particles	MRI imaging	Blood-pool contrast with longer lifetime	(189)
USPIO-PEI	MRI imaging	Determining nanoparticle vehicle unpackaging for gene	(190)
PEGMnCaP NPs	MRI imaging	PH-activatable contrast in cancer	(191)
Mn-nanotexaphyrin	MRI imaging	Imaging lymph node	(192)
Micelles with PTX and SPIO	MRI imaging	Delivering drug and MRI imaging	(193)
TF-biotinylated perfluocarbon-(Gd-DTPA-BOA)@(doxorubicin/paclitaxel)	MRI imaging	Evaluating and quantifying drug delivery system for vascular restenosis	(194)
FibPep-ION-Micelles	MRI imaging	Detecting and imaging thrombus	(195)
P-selectin-MNP(iron oxide)-PBP	MRI imaging	Imaging poststroke neuroinflammation	(196)
Mn-SPIO micella	MRI imaging	High power liver imaging contrast	(197)
TMADM-03	MRI imaging	Imaging pancreatic islet graft	(198)
DHCA functioned IONP labeled hMSCs	MRI imaging	Imaging and tracking stem cells	(199)
TCL-SPION-Apt	MRI imaging	Imaging prostate cancer cells and chemotherapy	(200)
^18^F-labeled DBCO-PEGylated MSN	PET	Imaging tumor	(201)
^125/124^I-labeled anti-ICAM-1/PVPh-NP	PET	Detecting pulmonary inflammation	(202)
^64^Cu labeled IT-101	PET	Monitoring pharmacokinetics and tumor dynamics	(203)
^64^Cu labeled CANF-comb nanoparticle	PET	Imaging natriuretic peptide clearance receptor in prostate cancer	(204)
^64^Cu-TNP	PET	Imaging macrophages in inflammatory atherosclerosis	(205)
^64^Cu labeled CLIO-VT680	PET	Detecting rejection and immunomodulation in cardiac allografts	(206)
^64^Cu labeled CANF-comb nanoparticle	PET	Imaging atherosclerosis in artery	(207)
^125^I silver nanoparticle	SPECT	Monitoring distribution of nanoparticles	(208)
^125^I labeled cRGD-PEG-AuNP	SPECT	Detecting cancer cells and imaging tumor sites	(209)
^111^In labeled lipid/calcium/phosphate NPs	SPECT	Imaging lymph node metastasis	(210)
^111^In-MSN labeled neural stem cells	SPECT	Tracking glioblastoma	(211)
PSMA-specific aptamer conjugated AuNP	CT	Imaging prostate cancer cells	(212)
Liposomal iodine	CT	Imaging macrophage-rich atherosclerotic plaques	(213)
Liposomal-iodine	CT	Identifying tumor vascular structure	(214)
Tantalum oxide	CT	Producing greater imaging capability than iodine	(215)
AuNP	CT	Incorporating RBC to image blood flow	(216)
AuNP	CT	Labeling tumor cells to image tumor growth	(217)
AuNP	CT	Imaging brain malignant gliomas and enhancing radiotherapy	(218)
AuNP	CT	AuNP with CT contrast capability	(219)
Liposomal iodine	CT	Imaging tumor	(220)
AuNP	CT	Tracking mesenchymal stem cells	(221)

### 1.4 Incorporation of Multiple Imaging Agents in Radio-Nanoparticles

**Figure 5:**
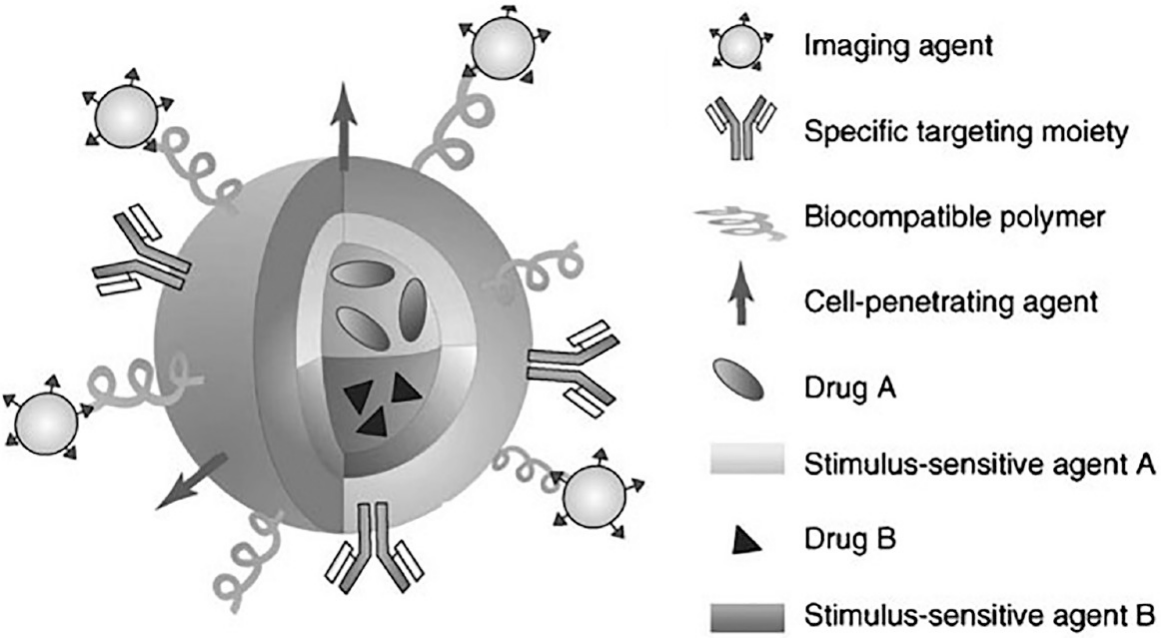
Representation of a nanodimensional synthetic platform suggestive of the multifunctional possibility of nanomedicines. Image reproduced with permission from ref (70). Copyright 2009 John Wiley and Sons.^70^

There are several types of imaging modalities that can be used to noninvasively detect a variety of biological processes, but there are limitations to their abilities to describe biological phenomena *in vitro* or *in vivo*. By combining techniques in multimodality probes it would be possible to overcome the limitations of a single-modality probe (Figure 5).^70^ For example, fluorescence imaging is a convenient technique; however, it is difficult to obtain high quality *in vivo* images due to high autofluorescence backgrounds. In this sense, bioluminescent proteins are therefore favored for *in vivo* imaging so far, as there is no high autofluorescence background; however, with this technique tissue attenuation becomes a serious problem for depth imaging. MRI/PET is easily able to overcome these depth-attenuation problems especially when involving dual nanoparticles decorated with long-lived radioisotopes such as ^64^Cu.^71^

As touched upon above, MRI provides good spatial resolution, but its sensitivity does not match its resolution capabilities. PET on the other hand has excellent sensitivity but poorer resolution, when compared to MRI or optical imaging.^72^ It has been found that paramagnetic metal cations, such as gadolinium or dysprosium, or superparamagnetic nanoparticles make particularly good contrast agents.^73−76^ Also, Chen et al. have combined PET/MRI in a dual modality probe to gain the resolution of MRI and the molecular/functional information the technique can provide and the sensitivity of PET and the anatomic/functional information it can obtain.^77^

Magnetic nanoparticles with their biocompatibility and low clinic toxicity are the perfect platform for other imaging techniques, while the magnetic element provides another imaging modality in the form of MRI.^78^ Devaraj et al. have developed an ^18^F trimodal nanoparticle, which combines MRI, PET, and fluorescence imaging techniques.^78^ Kim et al. have promoted the concept of multimodality probes and synthesized a quadruple imaging probe, ^68^Ga-MNP@SiO_2_ (RITC)-PEG/NH2-Fluc, a “hyphenated” radio-nanomedicine best described as a magnetic and fluorescent-bioluminescent-radioisotope-labeled particle.^72^ Interestingly, multimodal nanoparticles suitable for SPECT/PET combinations, which are biocompatible and can be designed to incorporate a large number of chelators and other functional groups such as targeting ligands, thus ensuring a high imaging signal strength and targeting capabilities, were reported. Tailored bisphosphonate-decorated and PEG-ylated superparamagnetic nanoparticles based on iron oxide proved to be long-circulating on the basis of their functionalized surfaces that controlled their colloidal properties as well as relevant for multimodal SPECT-MRI (T1 modality).^79^

Chen and co-workers^80^ reported a near-infrared fluorescence (NIRF) labeled high-density lipoprotein (HDL) nanoparticle (Figure 3b) to assess both active specific targeting to blood vessels in tumors and passive accumulation (due to the enhanced permeability and retention effect). The results showed that nanoparticles functionalized with a specific targeting system accumulate immediately after administration, while passive targeted accumulation of a nonspecific probe is the main event over a longer time. This study gives an insight into how the OI can be used for the kinetic assessment of drug accumulation in tumors.

The possibility to exploit OI for detecting alterations in the tumor environment has been reported by Kim et al.^81^ by assessing the accumulation of a hydrocyanine-labeled nanoparticle (Hydrocyanine-NC) in subcutaneous mice xenografts. The principle behind this study is that tumors are often involved in inflammatory and immune responses that are characterized by an increase of reactive oxygen species (ROS). In the presence of such an oxidative environment, the hydrocyanine moiety undergoes an oxidation reaction that produces a fluorescent cyanine dye. Hydrocyanine-NC could develop strong fluorescence intensity in a dose-dependent manner of ROS, and it showed strong intracellular fluorescence after treatment of macrophage cells (RAW 264.7) with the cytotoxic agent lipopolysaccharide (LPS), whereas in nontreated cells no fluorescence image was obtained.

Nanoceramics, a widely used class of inorganic particles of less than 100 nm diameter, are formed by metal oxides including silica nanoparticles and are frequently obtained by controlled sol–gel processes. There is an increased interest in the applications of nanoceramics in biomedical applications due to their excellent modulable properties, which is the case with most of the silica-coated/metallic NPs involved in multimodal imaging applications discussed hereby. In this regard, ceramic nanoparticles are emerging as potential candidates for medical imaging agents.^82−84^

## 2 Radiolabeled Nanoparticle-Based Agents for Tracing Hypoxia

**Figure 6:**
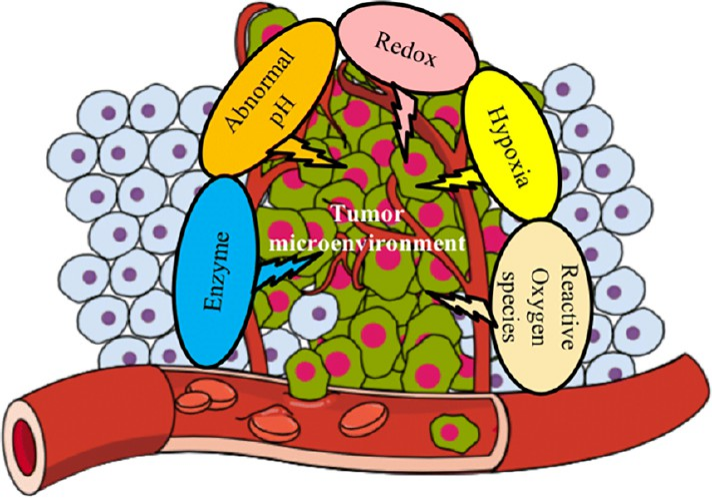
Representations of a malignant solid tumor with its different areas depicting deregulated pharmacology. Image reprinted with permission under a Creative Commons [CC-BY 4.0] from ref (99), an Open Access article distributed under the terms and conditions of the Creative Commons Attribution (CC BY) license. Copyright 2018. [2018 by the authors].

Hypoxia is a microenvironment condition characterized by decreased oxygen content at the cellular level. This particular condition is present in the microenvironment of many tumors, due to an imbalance between the quantity of oxygen available and its enhanced consumption rate by cancer cells.^85^ Another cause has been assigned to the physiological characteristics of the tumor microvasculature, which is often undeveloped, thus limiting oxygen diffusion in deep tissues (Figure 6).

Over the years, significant efforts have been employed in addressing challenges in hypoxia targeting with small molecular tracers, and we, and others, have highlighted the unmet research needs in the field over the years.^9,11−21^

Tissues hypoxia is seen as a central factor for tumor aggressiveness and metastasis, independent of factors such as tumor stage and nodal status.^86,87^ Hypoxic areas appear as a result of a disequilibrium between the supply and consumption of oxygen. Areas with O_2_ tensions (*p*O_2_ values) ≤ 2.5 mmHg are considered hypoxic tissue areas. These areas can be found in a broad variety of human malignancies, e.g., breast, uterine, cervix, head and neck, prostate, rectum and lung cancer, brain tumors, and malignant melanomas.^87−96^ Hypoxia starts at a very early stage, during tumor development from a tumor diameter of just a few millimeters.^86,97,98^

There has been growing interest in developing noninvasive cancer treatments that can target hypoxic tumors; however, there remain many important questions to answer to develop the long-standing goal of exploiting tumor hypoxia as the best validated target in oncology.^99^ Noninvasive assessment of tumor hypoxia via imaging techniques is possible like with PET or SPECT by detection of radiolabeled tracers or with MRI techniques. However, clinical experience using these methods in patients is so far very limited.^87^ The state-of-the-art depicts a range of representation of malignant solid tumor with its four different areas. The tissue area next to the blood vessel is normoxic, as the supply and consumption of O_2_ is “normal”, as it should be in most cells. The next area is hypoxic, having a deficit of O_2_. Finally, the last area is necrosis, which corresponds to the dead cells. Therapy resistance is greater in necrosis and hypoxic areas, and O_2_ and nutrients arrive less effectively at these areas. PET imaging gives quantitative information about hypoxia distributions in specific regions. The doses of the radiotracers that are injected are between nanomolar and picomolar concentrations, and as a result minimum side effects are produced in biological cells and the degree of hypoxia is merely reported. To determine the presence of tumor hypoxia, a hypoxic selective agent is required. A hypoxia selective agent needs to have a high membrane permeability to allow for easy access to intracellular mitochondria, and it has been demonstrated that an intrinsically biologically accessible redox potential is necessary. In addition, PET informs on tumor formation with repeated and quantifiable measurements. PET has unique advantages and is preferable for clinical imaging of hypoxia tumors because it has a high target-to-background contrast ratio and high resolution for tomographic imaging.

Small-molecular tracers for hypoxia emerged with the development of 2-nitroimidazoles as hypoxic cell radiosensitizers, and these were the first generation of molecular probes for PET. It has been demonstrated that small molecular species based on 2-nitroimidazoles localize in tumors and their reduction occurs in hypoxic environments. This reduction requires the presence of active tissue reductases, which exist in hypoxic cells while their accumulation also needs to take place in hypoxic cells instead of normoxic or necrotic cells. Otherwise, into normoxic cells reoxidization of nitroimidazoles causes diffusion of the cell and, as a result, selectivity. ^18^F-FMISO is the prototype tracer of 2-nitroimidazole which is used in PET diagnosis of hypoxic tumors. The lipophilic structure ensures cell-membrane penetration and diffusion into the cell, and some studies mentioned that ^18^F-FMISO is used for the direct oxygen measurements in hypoxic tissues. However, it is not generally acceptable for clinical use because of the slow pharmacokinetic profile, which is limited by the normal tissue clearance. Then, ^18^F-FAZA, the more hydrophilic analogue of ^18^F-FMISO, was introduced for PET diagnostic purposes, as it has faster clearance kinetics. This was shown to improve the ratio of tumor to reference tissue and hypoxia to normoxia contrast. The higher tumor to reference tissue ratio of ^18^F-FAZA makes it a potent tracer for clinical applications. Another example is ^18^F-FRP-170, which has shorter interval before the scanning and improved the hypoxic contrast, thus making it potent to use in clinical tests.

Copper-labeled diacetyl-bis(*N*-methylthiosemicarbazone) (Cu-ATSM) is another small molecular tracer that images hypoxia as a PET radiopharmaceutical, when labeled with ^64^Cu (Figure 3c). Cu-ATSM has high cell membrane permeability and diffuses readily from the bloodstream to surrounding cells because is a neutral lipophilic molecule with low molecular weight. It reduces in hypoxic cells and is entangled within them, although this does not happen in normoxic cells, where it is washed out without any change. The intracellular reduction of Cu(II) to Cu(I) and the reoxidation by intracellular molecular oxygen is currently believed to be involved at the origin of the hypoxia specificity of Cu-ATSM. Additionally, the radioisotope [^64^Cu] enhanced DNA damage and cytotoxicity in hypoxic cells.^42^ In contrast with other hypoxia tracers, ^64^Cu-ATSM has several advantages such as simple synthesis/radiolabeling methodology and faster clearance from normoxic cells. Faster clearance allows shorter intervals between injection and imaging and higher hypoxic to normoxic contrast.^42^

Self-assembled nanoparticles are utilized to treat hypoxia, which causes various intractable diseases, by the selective release of the hydrophobic agents under the hypoxic conditions. The main content of these self-assembled nanoparticles is amphiphilic polymers, which are mentioned as nanocarriers for anticancer drugs. The characteristics of amphiphilic polymers are drug solubility, high thermodynamic stability, and preferential accumulation in tumor tissue. In terms of probe design elements, hypoxia tracers incorporate lipophilic functionalities, that help to enter the cellular environment and uniform cell distribution, as well as hydrophilic tags, to avoid membrane sequestration and faster cleaning by normoxic cells and systemic circulation. Parameters such as blood flow or pH partially affect the pharmacokinetic profile and tissue distribution in hypoxia. In *in vivo* assays, tracers should be stable against nonhypoxia metabolism, and in clinical tests, the tissue kinetics could have the permission of time frame for the imaging. Finally, the radiotracers should affect a large variety of tumor types, be easy to synthesize, be readily available, have an amenable dosimetry profile, and reproducibly detect hypoxia.^42^

In terms of radiolabeled particles, very recently nitroimidazoles were incorporated onto Au NPs that simultaneously featured a chelator, for targeting hypoxia in cells. This new hypoxia-targeting platform may be of relevance to imaging hypoxia, as well as in a multimodality scenario for the delivery of a therapeutic dose of radiation or radiosensitizers additionally to the possibility of delivery of chemotherapeutic drugs to hypoxic cells. The incorporation of the bioreductive marker, 2-nitroimidazole (a small molecule hypoxia-homing molecule, as noted above, which can undergo selective oxygen-dependent reduction in hypoxic cells), was necessary to ensure hypoxia selectivity. Additionally, the surface of the AuNP was decorated with a versatile bifunctional chelator, DOTAGA, known for its ability to incorporate a range of metallic radioisotopes (such as lutetium-177, yttrium-90, or gallium-68). While CHO cell uptake studies under hypoxia were promising, interestingly, when the biodistribution studies of this new hybrid (denoted [^177^Lu]Lu-DOTAGA-AuNP-2-NIM) were carried out with Swiss mice bearing fibrosarcoma tumors, the apparent tumor uptake was minimal. However, the fast clearance of these nanoparticles *in vivo* was demonstrated (ca. 70% of the injected radioactivity was excreted within 3 h of injection).^100^

This observation was in line with similar findings for Au NPs labeled with gallium-67-labeled bombesin-conjugated gold nanoparticles of similar characteristics (but which were not designed for hypoxia targeting).^101^

## 3 Design and Functionalization of Nanomedicine Scaffolds

Nanomedicine can be defined as a new branch of research where the applications of nanotechnology are applied to medicine and the delivery of drugs to specific targets. The field of nanomedicine differs significantly in comparison to conventional therapy in that it aims to destroy specific cells or repair them one cell at a time rather than just attempting to remove diseased cells faster than healthy ones. The implication of nanomedicine for society is that it contributes to the possibility of personalized medicine. This already represents a significant paradigm shift in medicine, and the hope is that it will allow medical professionals to advise each patient on the most suitable pharmacotherapy based on individual profiling. A move to personalized medicine is desirable because it should decrease adverse drug reactions in patients including side effects and overdoses, as well as improve the efficiency of treatment of many diseases.

**Figure 7:**
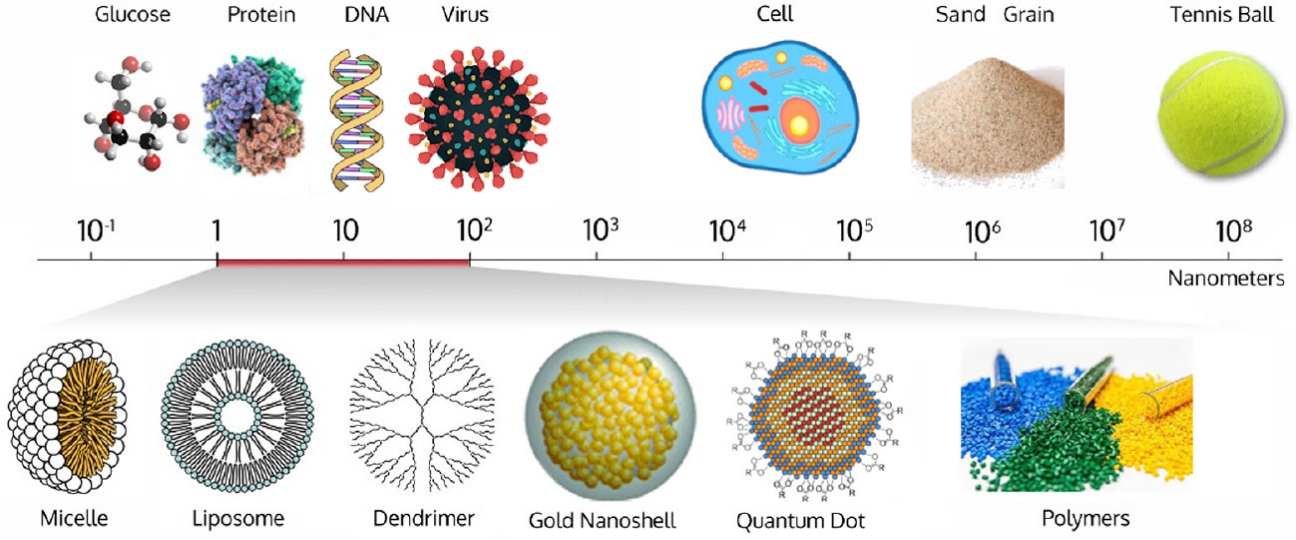
Overview of different categories of nanocarriers relevant as synthetic materials scaffold for radionanoparticulate drug delivery and their relative size.

The nanomedicine design and development^99^ focused on a large variety of nanocarriers such as liposomes, micelles, dendrimers, polymers, carbon nanotubes, quantum dots, iron oxide, gold nanoparticles, and mesoporous silica (Figure 7). Core–shell nanoparticles have multidirectional applications, are used for long-term combined therapy, and have revolutionized the efficacy of diagnostic nanomedicines. Moreover, core–shell nanoparticles can be applied on a cellular level and molecular scale and, as such, represent a promising approach as synthetic scaffolds for nanomedicines.^9^

### 3.1 Cancer Application-Driven Design of Nanomedicines

Over the past two decades, clinical research included the use of nanotechnology in medical applications with an aim to better understand and treat prevalent human diseases, especially noncommunicable diseases (e.g., cancer). This application, better known as nanomedicine, is an innovative and exciting area of science that has been intensively researched with excellent results, showing the huge potential of nanomedicine in disease diagnosis and therapy. Therefore, the development of drug delivery systems, health monitoring, as well as disease diagnostics and screening are major areas which have been researched to help revolutionize medicine and achieve this goal.^102^ The advances being made in nanomedicine show wide potential for innovative precision medical devices and the ability to develop the specific treatments required, resulting in tailor-made therapeutic options for each patient.^103^

**Figure 8:**
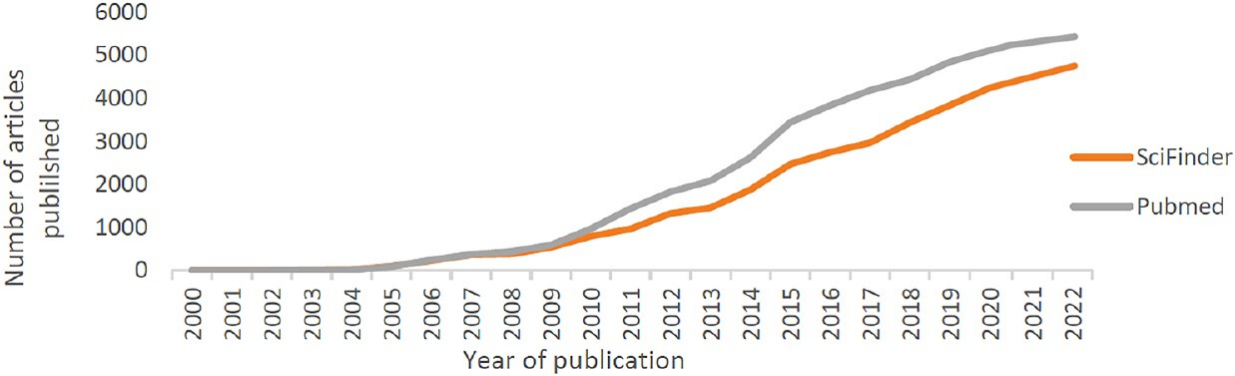
Trends in the number of published articles with keyword “nanomedicine” as emerging from PubMed and SciFinder overview of the decades 2000–2022.

Cancer has been identified as being the second leading cause of death globally, causing 1 in 6 deaths. Therefore, cancer prevention, detection, and treatments are critical issues worldwide and need a great deal of research over the following years to try to minimize the incidence globally. While survival rates for cancer diagnoses are much better than they once were, there is still poor prognosis for several types of cancer, such as brain, lung, liver, and esophagus, which only have a 25% chance of 5-year survival. Furthermore, those living with cancer and those that have survived have been estimated to have 1 in 4 people living on a long-term basis with at least one physical or psychosocial impact brought on by either their cancer diagnosis or treatment of their cancer. In April 2021 there were a total of 86 clinical trials for the application of nanotechnology in cancer treatments worldwide according to the U.S. National Library of Medicine (NIH) (Figure 8) (Search of: nano | cancer - List Results - ClinicalTrials.gov). Main-stream scientific article publishers and databases such as SciFinder and PubMed show the drastic increase in interest over the past decade.

### 3.2 The “All-in-One” Approach for Radio-Nanomedicine Design

Current progress in bioinspired fabrication approaches to nanomedicines and their radioactive analogues incorporated aspects of nanomaterial self-assembly and elements of molecular recognition and soft matter chemistry in synthesis and analytical characterization, aiming to develop sustainable and scalable methods for functional materials suitable for healthcare application and batch-to-batch reproducibility with nanometer-level precision. Such approaches opened the door to more accessible, large-scale, and sustainable production of future nanomedicines, exploring the possibility for manufacturing adoption by the pharmaceutical industry. Challenges in design and assembly of radio-nanomedicines remain especially in terms of their environmental footprint and the fact that complex nanoparticulate hybrids of relevance to biomedical applications often encounter difficulties in scaled-up processes. As such, molecular-level control has been hailed as a significant advancement in the design of nanomedicines aiming to facilitate the selective binding of biological materials to inorganic substrates.

Molecular imaging therefore plays a key role in personalized and targeted medicine.^84^ Among the imaging modalities for cancer diagnosis and treatment, fluorescence imaging, positron emission tomography (PET), and single-photon emission computed tomography (SPECT) have gathered considerable research interest, including in the realm of radio-nanochemistry approaches to probe design. The main advantages of optical imaging compared with other imaging modalities are superior sensitivity, low energy radiation, the capacity to monitor multiple independent optical biomarker reporters simultaneously, and relatively simple imaging hardware.

There is a necessity of developing highly sensitive imaging tools that involve the medical applications of luminescent nanoparticles, enabling highly sensitive *in vivo* optical detection. This is mainly due to the possibility that nanoparticulate scaffolds used as diagnostics would allow the concentration on their surface of a wide selection of sensing and imaging molecules with adequate properties to provide a good signal that can be exploited to image a variety of noncommunicable diseases, especially cancers.^104^ However, despite remarkable accuracy and considerable versatility toward identifying instances of cancer, these technologies still present a very low sensitivity. and there are also issues associated with incidental findings that can complicate the interpretation of resulting images.^12,105^ In addition, to date, there are no widely accessible cases of incorporating simultaneous therapeutic strategies within MRI imaging into the clinical praxis, and there is no accessibility to early noninvasive diagnostics of cancer in wider communities.

Fluorescence or photoluminescence techniques, reliant on photons as the energy source,^106^ remain the most widely used in biopsy diagnostics; additionally, fluorescent imaging can be used to track and evaluate the efficiency of the drugs release and complementary to photodynamic therapies. Optical imaging techniques applied to date in diagnostic biopsies as well as in life sciences assays employ a number of well-established organic molecules further functionalized in order to be directed to target cancer specifically, such as Rhodamine, derivatives of fluorescein, and more recently near-infrared (NIR)-emitting cyanine dyes.^84^ New NIR absorbing and emitting nanoprobes for advances in single- and multiplexing arrays used in biosensing technologies are the “Holy Grail”, yet challenges remain regarding the materials synthesis: bath-to-batch reproducibility, size and shape control, biocompatibility when loaded into cells, as well as the bio- and photophysical characterization using microspectroscopy and imaging techniques.

Nanomedicines can incorporate and deliver more than one bioactive molecule, thanks to their large surface areas that can be easily functionalized due to the silica or similar encapsulating nanoceramics. These bioactive molecules can include targeting and therapeutic agents and image contrast enhancers. However, challenges remain in the assembly of biologically compatible systems, including radio-nanomedicines.

Core–shell particles or organic/polymeric nanoparticles including liposomes have advanced to the most promising level of acceptability for preclinical and clinical trials largely due to their most promising batch-to-batch synthetic consistency. An overall size of significantly less than 300 nm is generally required to ensure these materials can adequately bind to the biological species within cells without steric hindrance effects and can be used in low concentration such that they do not interfere with the system being tested. This requires that particles with highly controlled size distributions are produced and demands that novel nanoparticle manufacturing technologies are developed for both materials and the conjugated tags. Nanomaterial fabrication is challenging, especially for nanoceramics, as often the production of these materials involves high temperatures where their crystalline phases are tailored for ideal optical performance, while still ensuring the material is monodispersed and free of agglomerates.

In this context, certain key synthetic challenges in radio-nanomedicines assembly remain a subject of research interest:

- There is need to develop (nano)materials with superior performance over existing commercial or off-the-shelf nanomaterials, that enable facile and versatile radio-incorporations of diagnostic isotopes with differing half-lives and energy characteristics, as technical requirements to handle these will differ widely.
- There is a need to ensure batch-to-batch reproducibility in the production of core particles which are likely to render these biocompatible, e.g., with radius smaller than 100 nm. There is the emphatic need to demonstrate robust and reproducible surface chemistry compatible with the linking of biologically active molecules (as targeting groups) and ensuring tunable dimensions of the construct.
- For multimodality imaging probes, additionally to the incorporation of the radioisotope, it is important to retain the brightness of the fluorescent/luminescent tag such that photobleaching in solution and in cells invertedly affects their traceability on cells.
- Detailed and reproducible assays are needed to evaluate the cellular morphology upon treatment with nanomaterials as a first indicator of the degree of toxicity in live cell imaging requirements.

### 3.3 Metallic and Nonmetallic Nanoparticles As Synthetic Scaffolds for Nanomedicines

Nanoparticles have recently been introduced in “nanotheranostics”, with gold and iron oxide nanoparticles currently entering in clinical trials while a much wider range being available in preclinical *in vitro* and *in vivo* tests.

Recent studies show the improvement in pharmacokinetics gene therapy by assisting the progress of delivery into tumors and the crossing of complex biological barriers. Consequently, higher drug delivery, efficient-stimulus response toward the surrounding environment, and potential capability to target specific tumors is a result of the high surface area to volume ratio in nanoparticles.^107^

Nanoparticles can be divided into several different categories, and several classifications emerged. In terms of radio-nanomaterials scaffolds, the most common ones for preclinical aspirations are those that incorporate lipid-, polymeric-, and inorganic-based materials. Cationic liposomes are the most considerable invention of the lipid-based nanocarriers group. They are composed of cationic lipids and neutrally charged helper lipids. The latest interact with nucleic acids and create a lipoplex, which protects the liposome from enzymatic degradation in blood circulation and facilitates cell internalization by interacting with cell membranes. Cationic-based nanoparticles are organic nanoparticles for gene delivery. Their advantages are the small size, which contributes to the narrow distribution, the ability to encapsulate in a variety of gene therapeutics, which protects them against enzymatic degradation, tunable physicochemical properties, and excellent stability *in vitro* and *in vivo*. Inorganic nanoparticles include carbon nanotubes and are used in gene delivery. More examples of inorganic nanoparticles are magnetic, calcium, phosphate, gold, and silica nanoparticles, with diverse morphologies, and these were classified according to their size, shape, composition, and chemical properties.^9,21,108^

Nanoparticle properties determine their biodistribution, their interaction with cell components, and the formation of a protein corona. Moreover, physical and chemical properties of nanoparticles correlate with the drug loading capacity, colloidal stability, and interaction with loaded drugs. The most important property is the shape, which affects size distribution and then the charge with the effectiveness to the stability and size distribution.^109^ Interestingly, the low dose levels of the toxic agent are directly correlated with the toxicity levels in the body. Many studies show that the surrounding environment influences the properties of nanoparticle formulation.^110^

Advantages of employing nanomedicines compared to “traditional” diagnostic and therapeutic methods include the following:

- It has been shown that the application of NP in molecular imaging differs considerably from the role of single molecular species: this is because these can easily integrate more than one kind of imaging or therapeutic agents, e.g., fulfilling a role as multifunctional nanoplatforms for both diagnosis and therapy. This permits the variation of the synthesis parameters and enables judicious modification of the size and aqueous media dispersibility, for example, leading to emerging functional core–shell nanoparticles.
- Nanoparticles exhibit large surface area/interior cargo volumes, and as such, some considerable numbers of imaging agents or drugs can be hosted within or on the surface of NPs through noncovalent incorporation and/or chemical conjugation.
- The specific targeting moieties or physicochemical optimization of size and surface properties can be carried out in detail, and as such, NPs can target disease sites for drug delivery and imaging.
- NPs can include more than one targeting molecule: this can greatly enhance target-binding and specificity compared to single molecules, due to so-called multivalent effects. However, aspects of kinetic stability and probe integrity/reproducibility remain challenges to be addressed in the design elements.
- appropriate size and surface modification of NPs can lead to enhanced circulation time in the blood reducing opsonisation and uptake into the reticuloendothelial system (RES).^111^

**Figure 9:**
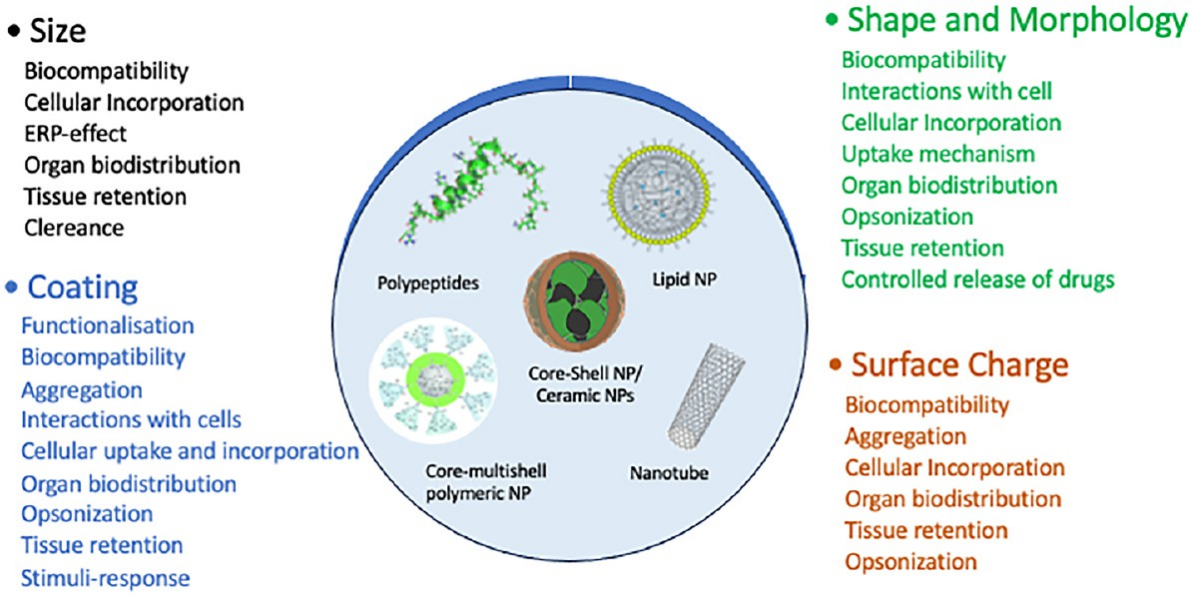
Overview of selected nanoparticles and their main properties and characteristics applicable to theranostics design.

Overall, it has been highlighted that the most important properties of nanomaterials determining their theranostic potential are (1) coating; (2) size; (3) morphology; and (4) surface charge. In terms of radio-nanomedicine assemblies or multimodality nanotheranostics design, the ability to incorporate a large and differing number of radioactive units with different half-life characteristics could constitute a further advantage (Figure 9).

The surface modification of nanoparticles, i.e., the nature of the surface coating, is crucial as nanoparticles start interacting with the biomolecules as soon as they enter the body. The interface transformations and related processes at the organic- or inorganic–biological boundary *in vivo* and *in vitro* is especially relevant for nanomaterials due to their higher surface-to-volume ratio. It is widely appreciated that in living systems proteins adsorb onto the surfaces of nanoparticles of all types and morphologies, forming a corona. Upon coating with this corona, the original nanoparticles’ surface gains further biological characteristics, entirely different to those of bulk materials: therefore, their *in vivo* performance can also become very different from what was originally envisaged from the perspective of the inorganic or organic surface chemistry employed at design stage.

**Figure 10:**
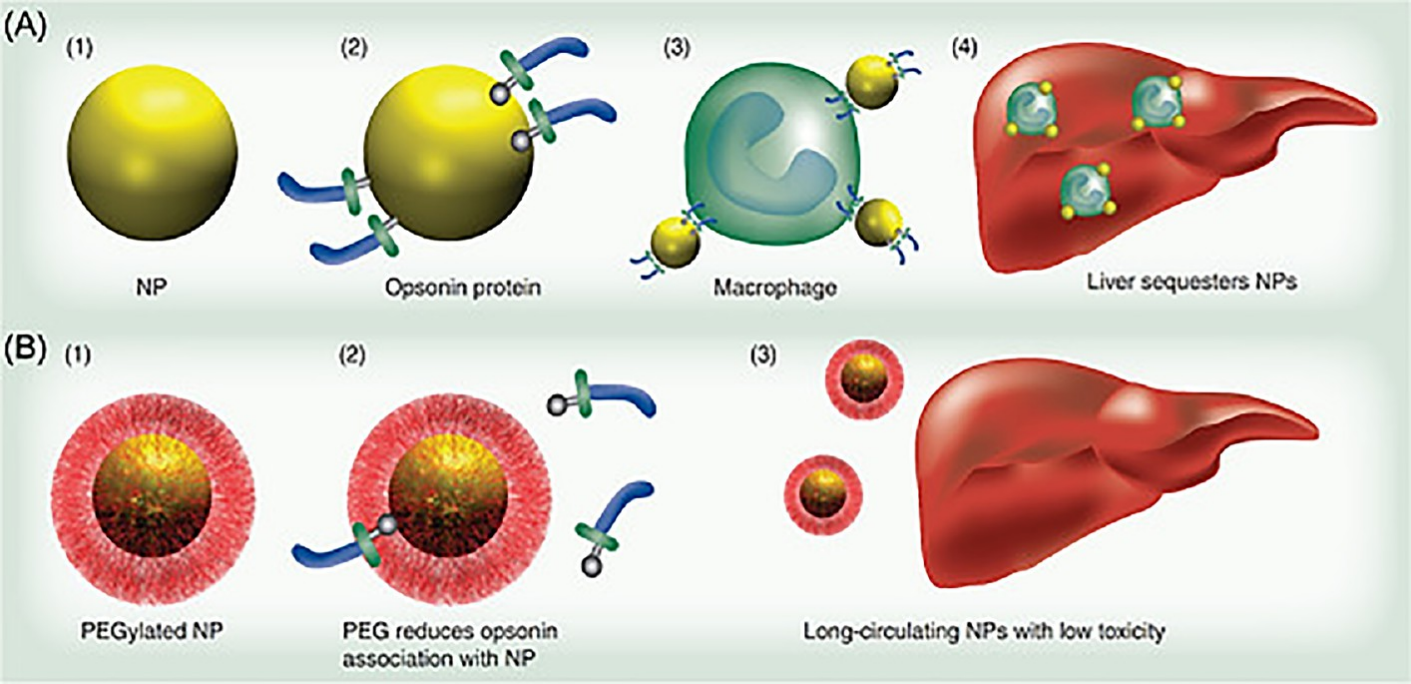
Polyethylene glycol prevents uptake by the reticuloendothelial system. (A) Nanoparticles (NP) (A1) are coated with opsonin proteins (A2) and associate with macrophages (A3) for their transit to the liver (A4). Macrophages stationary in the liver, known as Kupffer cells, also participate in nanoparticle scavenging. (B) Nanoparticles coated with PEG coating (B1) prevents this opsonization (B2), resulting in decreased liver accumulation (B3) and increased availability of the NP for imaging or therapy. NP: Nanoparticle; PEG: Polyethylene glycol. Reproduced with permission from ref (112). Copyright 2018 Elsevier.^112^

This coating process is relevant to uptake as well as the circulation of nanoparticles, as it can help the immune cells (present either in blood circulation or tissues) to recognize the nanoparticles and thus mediate their uptake in the widely investigated process called opsonization.^111^ This process is highlighted in Figure 10.^112^

To ensure that engineered nanoparticles can circulate long enough *in vivo* (and reach the target tissue at the effective concentration, avoiding disintegration and morphological changes, or elimination caused by opsonization before their reach the target), design elements that can ensure the kinetic stability *in vivo* need to be considered. This is particularly relevant for the case of diagnostic radio-nanoparticles design as their *in vivo* degradation before an image is collected is particularly detrimental for the success of the radio-nanomedicine. It has been shown that polymeric coatings incorporated into the nanoparticle design can protect them against blood proteins (opsonins, in particular) and mediate their interactions with the immune system. As such, the most widely used oligomers/polymers are those based on polyethylene glycol (PEG) which can provide a highly hydrated shell (2–3 water molecules per monomer). This shell was deemed necessary to prevent the negative impact of the interactions between the nanoparticles of interest and biomacromolecules such as opsonins *in vivo*. The incorporation of this polymer has been FDA-approved for use in various drug formulations, for a wide range of therapeutic and diagnostic nanomedicines including for liposomes or iron oxide nanoparticles in clinical trials (Figure 10).^112^

## 4 Challenges for the *In Vitro* Delivery and Molecular Imaging with NPs

Generally, nanoparticles are covered with layer of polymer drugs, fluorophores, proteins, peptides, and oligonucleotides and then administered into cells (*in vitro*) and animals (*in vivo*). The interaction of serum proteins and cell membrane receptors with the nanoparticles influences cell uptake, gene expression, and toxicity. Ligand addition to the nanoparticles increases the selectivity to the receptors. The strength of the nanoparticle–ligand interaction based on the ligand density of nanomaterial and engineered geometry. Ligand binding affinity increases with the size of the nanoparticle owing to a higher protein density on the nanoparticle surface. One example is that the presence of Herceptin in gold nanoparticle conjugates with overall 40–50 nm size influences the caspase enzyme activation and alters cellular apoptosis. The peptide existence on the nanoparticle increases angiogenesis, which depends on receptor mediated signaling.^113^ Taking all this into account, the presence of the complex, nanoparticle–ligand, shows more advantages than if the free ligand was in solution.

**Figure 11:**
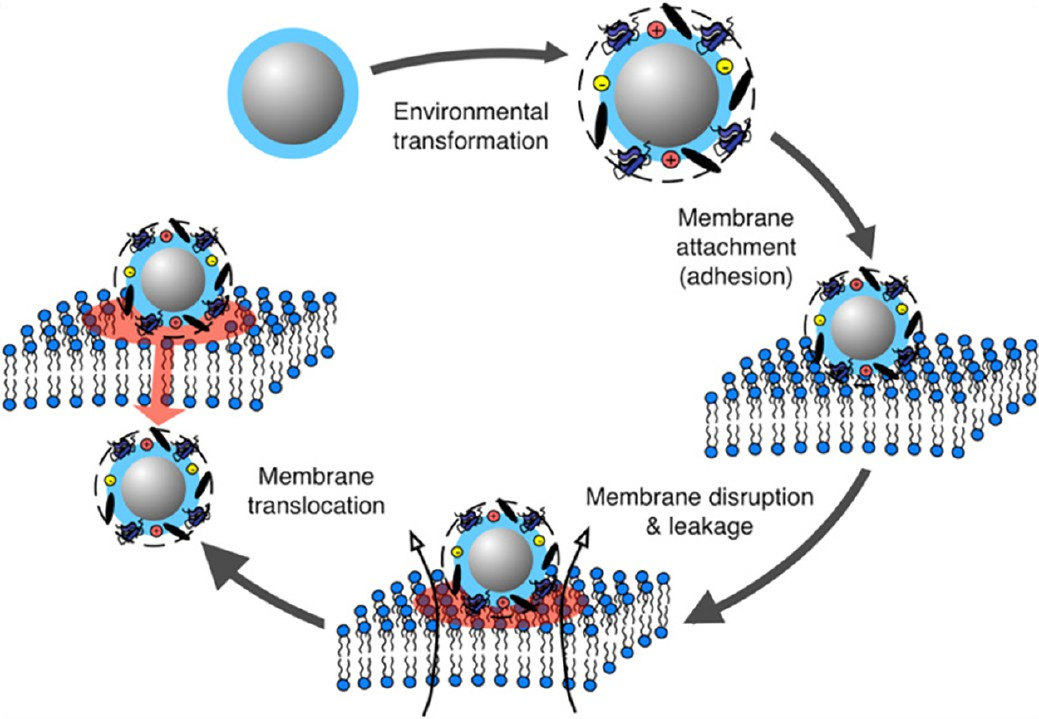
Nanoparticle interactions with cell membrane receptors, ultimately influencing delivery, mediated by size, shape charge of NPs, ligand density, receptor expression levels, internalization mechanism, and cell properties (phenotype, location, etc.). Image reproduced from ref (108).

The entrance of nanoparticles into the cell is affected by several factors such as shape, size, axis size, asymmetry, and composition of nanoparticles (Figure 11). Different shapes of nanoparticles show different uptake into the cell, preferably being spheres, cylinders, and cubes. Moreover, the nanomaterial’s dimensions relate to the cell uptake, as the maximum rate of uptake is achieved by spherical nanoparticles with 50 nm diameter. Likewise, the shape, size, and composition of nanoparticles affect uptake.

In general, nanoparticles show promising properties; they can be engineered to localize the specific site of a disease with lower doses and avoid the side effects that are associated with current methods of cancer treatment.^108^ Nevertheless, the limitations of synthetic routes for metal-containing nanoparticles limit the progression as a useful tool in cancer diagnosis and significantly limit their usefulness as radio-nanomedicine synthetic scaffolds. Finally, the main properties of nanoparticles need to include biocompatibility, low toxicity, lower clearance rates, the ability to target specific tissues, and controlled release of drugs.

Nanoparticle characteristics such as exposure route, concentration, and time can affect *in vivo* results. The dose for a specific tissue target is different by comparing *in vitro* and *in vivo* tests, as a result of the difference in nanoparticles kinetics, absorption, distribution, metabolism, and excretion (ADME).^114^ Consequently, the high cost of *in vivo* tests lead to uptake of *in vitro* models to test translocation of nanoparticles and estimate levels of internalization and *in vivo* effects.^114^

*In vitro* assays are useful to investigate the mechanism by underlying nanobiointeractions, evaluate for toxicity tests, risk assessment, and *in vivo* predictions. Moreover, they used to correlate the nanocarrier properties with the *in vivo* behavior and as a result reduce the number of animal and human trials. In the case of *in vitro* studies, the necessity to ensure nanocarrier stability, ability to fulfill the desired mission, and safety conformity is required. The temperature stability of NPs is crucial: *in vivo* tests should be performed at 37 °C to simulate body temperature. *In vitro* techniques are preferable due to better control of the experimental conditions, the ease of conduction, minimal ethical concerns, simpler interpretation of the obtained data, and inexpensiveness. However, the presence of biomolecules caused different interactions with nanocarriers than the expected ones. Furthermore, nanocarriers agglomeration often occurs *in vitro*/*in vivo*.

*In vitro* studies accompanied by *in vivo* tests using nanoparticles have been achieved the last 20 years, and these seem to suggest that neutral nanoparticles, which have lower blood half-life, are preferable instead of positively charged nanoparticles. In the case of using positive charge nanoparticles for *in vivo* tests, the complications are hemolysis and platelet aggregation due to the quicker response of nanoparticles instead of blood. Additionally, the positively charged nanoparticles interact with different types of proteins. For example, interaction between serum proteins and positively charged nanoparticles leads to the removal of the latter by the mononuclear phagocyte system (MPS). To avoid this clearance and ensure that nanoparticles continue to have their action, the most successful idea is the development of PEG to their surface. This addition increases the blood half-life of nanoparticles.

Llop et al. have recently described the current clinical landscape of radionuclide targeting, imaging, and therapy and reflect on the potential role of nanoparticles in these applications. They address the role that nanoparticles can play in these applications, highlighting the potential of nanoparticles for intraoperative imaging and, above all, for individualized and enhanced radionuclide therapy.^116^

### Size Matters and Addressing Brain Imaging Challenges

Commonly for *in vivo* tests, there are still unanswered questions about the modes of action of nanoparticles.

These questions include the nanoparticles unknown metabolism, the long-term fate of nanoparticles, and finally if the physicochemical properties of nanomaterials affect their biodistribution behavior *in vivo*. It is currently acknowledged that generally, nanoparticles enter the cell via (1) clathrin/caveolar-mediated endocytosis, (2) phagocytosis, (3) macropinocytosis, and (4) pinocytosis.

It is also generally accepted that nanoparticles exit the cellular environment via (1) lysosome secretion, (2) vesicle-related secretion, and (3) nonvesicle-related secretion.

Regarding brain imaging, there are a number of unmet clinical needs and challenges to be addressed: The blood–brain barrier (BBB) is a highly selective semipermeable membrane barrier that separates the circulating blood from the brain extracellular fluid in the central nervous system (CNS). The blood–brain barrier is formed by brain endothelial cells, which are connected by tight junctions.

**Figure 12:**
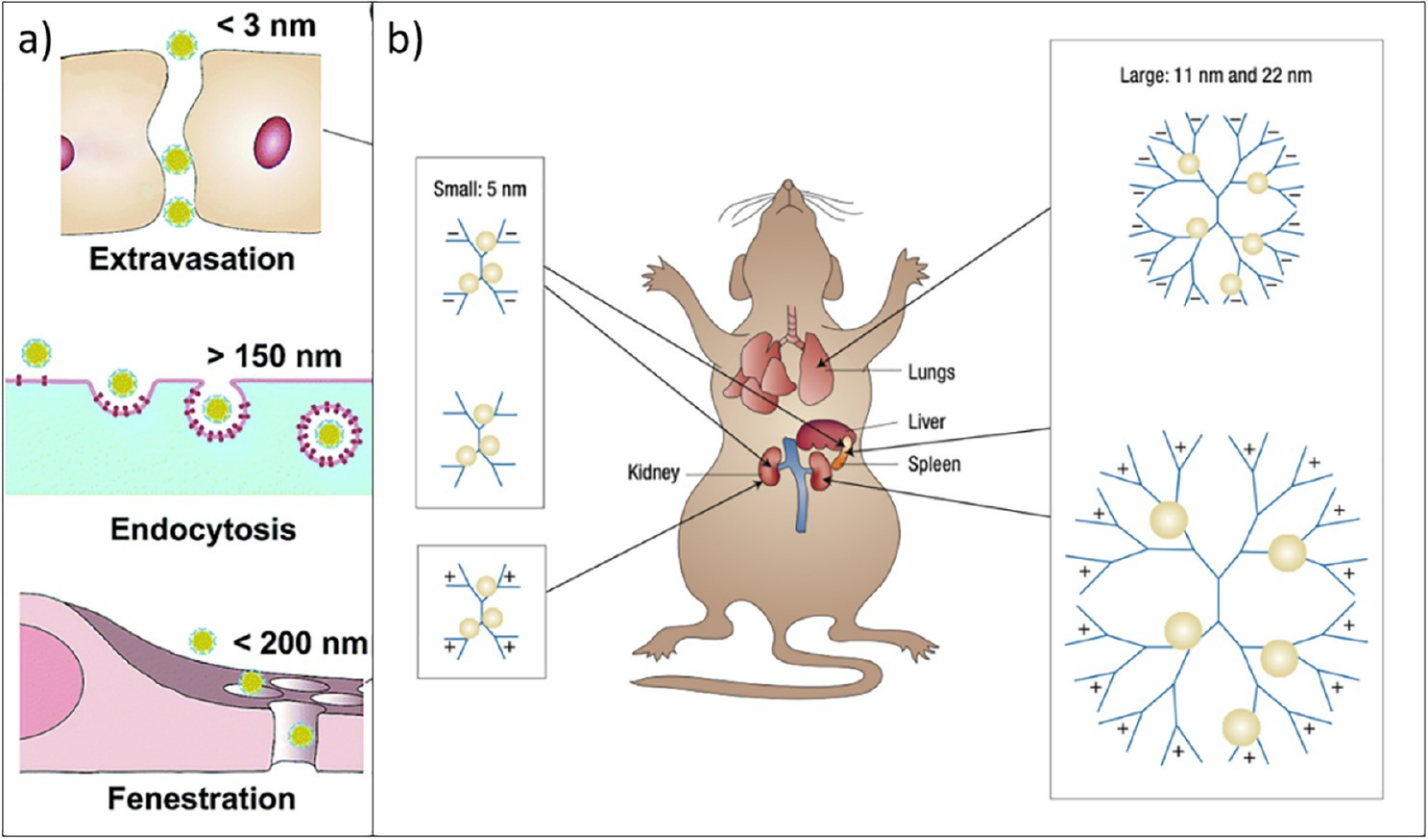
(a) Mechanisms of nanoparticle passive targeting: Nanoparticles smaller than 3 nm in diameter could extravasate different tissues nonspecifically. Nanoparticles with large negative surface charge or larger than 150 nm in diameter could be captured by Kupffer cells. Nanoparticles less than 200 nm in diameter could pass through sinusoidal fenestrations after intravenous administration^115^ and (b) gold–dendrimer nanoparticles and their biodistibution *in vivo*. Dendrimers are branched molecules that can be used as scaffolds for metals such as gold to attach to, enabling nanoparticles with different diameters and surfaces charges (left and right; – is negative charge, + is positive charge, and n is neutral) to be produced. Recent experiments show that the size and charge of the nanoparticles influence their biodistribution in mice.^115^ (Figure adapted with permission from ref (115). Copyright 2012 Royal Society of Chemistry.)

An early experiment by Michin^117^ showed that nanoparticles smaller than 3 nm in diameter could extravasate different tissues nonspecifically (Figure 12): nanoparticles less than 200 nm in diameter could pass through sinusoidal fenestrations after intravenous administration; <10 nm could cross the blood–brain barrier. Imaging showed that all the nanoparticles disappeared from the circulation with a half-life of 2 h or less.

However, for the different 5 nm particles, positively charged particles persisted in the kidneys; negative/neutral particles remain in liver/spleen. However, for a range of particles in the 22 nm which were also positively charged of low levels particles were found present in the kidney, and accumulation occurred in the lungs, liver, and spleen instead. Total urinary and fecal excretion after 4 days was greatest for the 5 nm positively charged nanoparticles. But, for 5 nm NPs less than 50% of the total dose was accounted for. Total excretion was much lower (between 6% and 15% of the total dose) for all the other nanoparticles, and the persistence of material in the tissues was indicated, which confirmed these observations.

Regarding delivery, these authors seem to suggest that particles that entered the peripheral tissues became either tightly bound or highly compartmentalized. This could lead to issues regarding longer-term exposure, and accumulation may lead to local tissue damage.

Interestingly, cross-linked dextran nanoparticles which were then further chelator-conjugated and ^89^Zr tagged have been shown to target macrophage response in tissues, aiming to shed light on the role of these cells in normal physiology or in disease models. Interesting, this study also showed that a size optimization of dextran nanoparticles needs to be performed for PET applications, and a range of nanoparticles between 2 and 30 nm showed size-dependent pharmacokinetics, renal clearance rates, and macrophage uptake *in vivo*. Consistent with previous work, the 5 nm nanoparticle showed considerable renal clearance, whereas the 13 nm nanoparticle had the highest level of macrophage uptake, which was desirable for the macrophage imaging reported.^51^

### Liposomes and Related Organic Nanoparticles as Radio-Nanomedicine Scaffolds

**Figure 13:**
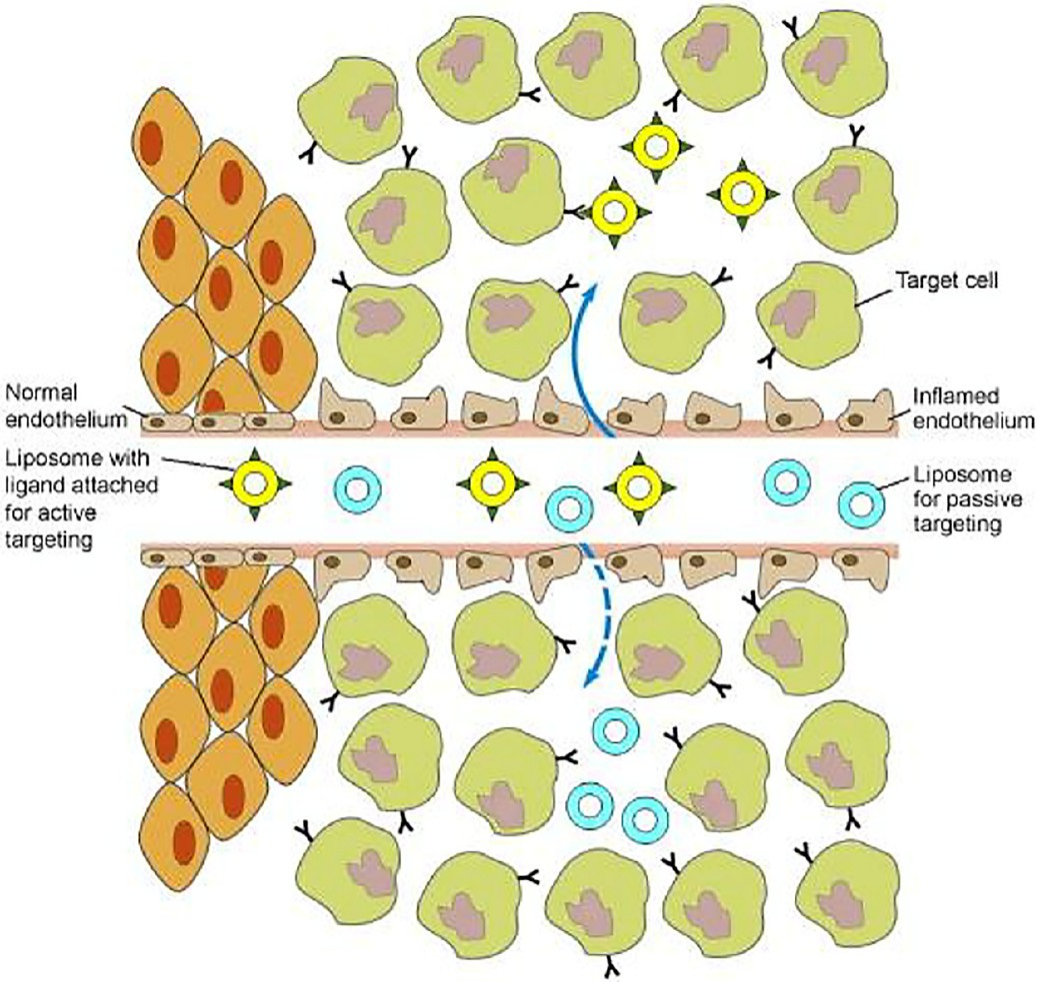
Active and passive targeting of nanoparticles (liposomes) to target cancer cells in chemotherapy. Reproduced with permission from ref (118). Copyright 2009 Elsevier.^118^

Liposomes are spherical structures with an aqueous core and a vesicle shell (Figure 13). Synthetic or natural phospholipids with cholesterol introduced in the bilayer membrane of the liposome help them to enter into the cell by endocytosis.^118,119^ Consequently, liposomes could easily cross the cell membrane because of the fusion of the lipid bilayer with the bilayer of membranes. Specifically, a hydrophobic region surrounds the aqueous solution that contains the drug (Figure 15). Furthermore, liposomes contain single or multiple bilayers, and this variation classifies them into three different categories: (a) multilamellar vesicles, (b) large unilamellar vesicles, and (c) small unilamellar vesicles.

Different ways that drugs can get into the liposomes are (a) liposome formation in an aqueous solution saturated with a soluble drug, (b) pH gradient methods, (c) the use of lipophilic drugs, and (d) the use of organic solvents and solvent exchange mechanisms.^118,119^

Since unmodified liposomes are rapidly cleared from the body by phagocytic cells, they are coated in a protective layer with a biocompatible (to prevent an adverse reaction in the patient) and inert (chemically inactive) polymer,^120^ such as polyethylene glycol (PEG). Liposomes also often have ligands attached, which match common receptors on cancer cells to promote active targeting. These nanoparticles may also make use of attaching photosensitizers to the outer layer for treatment therapies such as nanophotothermal and nanophotodynamic. Furthermore, solid lipid nanoparticles are very similar to liposomes, but instead of at least one bilayer of amphiphilic material, there is only a single layer of the phospholipids. The main difference in usefulness of liposomes and solid lipid nanoparticles depends on the production of the nanocarriers; therefore, solid lipid nanoparticles may become more readily available for general use and research. Polymeric micelles are much smaller versions of solid lipid nanoparticles and have an advantage in greater tissue-penetration capability.^121^ However, due to their small size, polymeric micelles can encapsulate less of the therapeutic drug in a single nanoparticle, and so more micelle nanocarriers would be needed than either liposomes or solid lipid nanoparticles to treat a tumor. Therefore, for treatment of a larger tumor, in which increased cancer therapy would be needed, liposomes or solid-lipid nanoparticles would be favored compared to polymeric micelles.

Liposomes have been of interest over the past few decades due to their ability to deliver anticancer agents in a manner that reduces the toxic effects of the drug itself, to increase the biocompatibility and kinetic stability of such a drug, and/or to increase the circulation time and effectiveness of the drugs. As such, these have also been considered useful in targeting multidrug resistance in cancer cases by reducing the chemotherapeutic efficacy. The proposed mechanisms to overcome multidrug resistance are (i) increase enzyme expression and especially expression of glutathione *S*-transferase, (ii) raise drug transporters and efflux proteins, and (iii) point the mutations in proteins that are targeted by drugs. Liposomes are biocompatible and biodegradable because of their ability to encapsulate hydrophilic agents in their core and hydrophobic agents into their vehicle and, as a result, become first-rate therapeutic agents. The insertion of polyethylene glycol (PEG) was shown to further improve the stability and circulation half-life of the liposomes.^51,119,122,123^

Several liposome-encapsulated or decorated radioisotopes have recently been reported. This is now a promising area of development considering that this technology has the potential to address issues of circulation *in vivo*, targeted delivery, as well as toxicity. These have already shown widespread interest for their therapeutic potential, and the theranostic approach where the molecular imaging aspects have been reported holds significant promise. Therefore, liposomal-based nanoparticles are versatile drug delivery vehicles as radio-nanotheranostics when labeled with long-lived radioisotopes such as ^52^ Mn (*t*_1/2_ = 5.591 days) and ^89^Zr (*t*_1/2_ = 3.3 days).^124,125^

### Core–Shell Nanoceramics and Metallic Nanoparticles as Radio-Nanomedicine Scaffolds

A range of nanoparticulate inorganic cores have been designed through the arrangement of different composite nanostructures, with the idea of combining two or more materials and thus different properties and functionalities within a single structure or geometry. Moreover, the surface modification of the inorganic structures with the organic counterparts becomes critical to increase functionality, stability, biocompatibility, and degree of dispersion and eventually providing the hybrid materials with extra functionalities and the intended multimodal nature.

This protocol aims to elucidate the luminescent properties of the nanocomposite *in vitro* and their organelle colocalization, internalization, and biological stability in living cells. This will allow the development of a new generation of hybrid organic–inorganic biomarkers for cancer detection and monitoring.^82,83^

Mesoporous silica nanoparticles (MSNs) are gaining increasing interest as the shell component of hybrid nanoparticles (e.g., as a crucial component of the core–shell entities with magnetic cores, or encapsulating inorganic oxides cores for biomedical applications): this important component of core–shell materials explored as nanomedicine components for drug delivery and specific labeling with fluorophores or radioisotopes. This ceramic layer acts as a biocompatible component in multifunctional nanomedicines to their several attractive features such as good biocompatibility, large surface area, tunable pore sizes, controllable particles sizes and shapes, and dual-functional surfaces (exterior and interior).^82,84^ The light transparency of the silica matrix enables the excitation and emission light to pass through the silica framework, as necessary for bioimaging applications. The chemical functionalization of such shells with targeting groups relies on the ability to incorporate bio-orthogonal linkers having the ability to attach “addresses” which in turn will tackle clinical needs for synthetic scaffolds appropriate as drug delivery systems for the biological imaging space.

Magnetic core–shell nanoparticles incorporate an encapsulated core or inner magnetic material and an outer shell composed of coating material, frequently mesoporous silica (e.g., a nanoceramic type material) or a soft organic polymer. The magnetic core and shell account for the magnetic and optical properties of these nanoparticles. The magnetic properties can be modified by the surface layer composition and the different elements that introduce it. Different categories of shells exist. Noble metals shells provide better biocompatibility, and they are resistant in the case of physiological changes, like a change in the pH. Moreover, noble metal shells do not allow agglomeration of the cores and are responsible for higher stability of the cores into different solvents, by keeping their properties unchanged.^9^ Additionally, magnetic oxide core–shells are used as fluorescence sensors through covalent bond formation with a fluorescent dye. Magnetic oxide core–shells include an inert surface of maghemite or magnetite. Another category is metallic magnet core–shell, with a magnetic core of metallic iron or cobalt within the inactive graphene shell. Comparing the last two categories, magnetic core–shell nanoparticles have many advantages such as (i) stable thermodynamically and superior chemical, (ii) accurate size distribution, (iii) more colloidal stable, (iv) magnetic moment depends on the nanoparticle cluster size, (v) direct covalent attachment by the silica surface, and (vi) preservation of superparamagnetic properties regardless of the cluster size of the nanostructures.^126^

The stability of metal complexes is a serious consideration in the biological environment due to the exhibition of high kinetic and thermodynamic stability, to avoid premature decomposition in living cells. MRI has been mentioned as the potential imaging modality of cancer tissue diagnosis because of using metal based nanoparticles. This relies on the ability of metal based nanoparticles to accumulate within the cells and increasing the signal-to-noise ratio for the higher resolution image of them.

In particular, ion oxide nanoparticles of relevance to biomedicinal applications generally have a diameter between 1 and 100 nanometers. The two main forms are magnetite (Fe_3_O_4_) and its oxidized form maghemite (γ-Fe_2_O_3_). They have attracted extensive interest due to their potential applications in many fields. Fe_3_O_4_ nanoparticles are known contrast agents currently of interest for magnetic resonance imaging (MRI), and they also have superparamagnetic properties.^127−129^

They are commonly used in a broad variety of therapeutic and diagnostic biomedical applications thanks to these properties, which enable tracking of theranostic nanomedicines by MRI. For *in vivo* applications, they can be administered intravenously into the body to detect and characterize lesions and tumors and to visualize body tissues. When used for MRI *in vivo*, iron oxide nanoparticles cause a critical decrease in the relaxation rate of water protons due to their high magnetization.

This enhanced contrast allows MRI to differentiate between different organs in the body and also between several tissues. Furthermore, iron oxide nanoparticles benefit from high chemical stability, low toxicity, and biocompatibility.^127,130^ Because of these attributes, iron oxide nanoparticles were chosen as the foundation for this multimodal system design and synthesis. They were therefore synthesized in the magnetite phase via a coprecipitation method.^130^ Among all the different methods of synthesis of iron oxide nanoparticles, this method appears to be most frequently applied due to its simplicity and ease in controlling the particle size.^131^ Iron oxide nanoparticles are well-known contrast agents for magnetic resonance imaging (MRI), and they also have superparamagnetic properties.^132,133^ They are used in a broad variety of therapeutic and diagnostic biomedical applications thanks to these properties. They can be administered intravenously into the body to detect and characterize lesions and tumors and to visualize bodily tissues. When used for MRI, iron oxide nanoparticles cause a critical decrease in the relaxation rate of water protons due to their high magnetization. This enhanced contrast allows MRI to differentiate between different organs in the body and between benign and malignant tissues. Furthermore, Iron oxide nanoparticles benefit from high chemical stability, low toxicity, and biocompatibility.^134^

Iron oxide nanoparticles found application in biomedicine because of their high biocompatibility and nontoxicity in humans: Iron oxide cytodiagnostics were tested in different solvents, such as water, as well as nonpolar environments.^62^

Aqueous iron oxide nanoparticles are used for the isolation and purification of proteins, DNA, viruses, and sometimes whole mammalian cells. The differentiation at the surface of iron oxide nanoparticles increases the biocompatibility and gives them a favorable pharmacokinetic profile. In addition, coated iron oxide nanoparticles have high specificity for a disease because of their new magnetic properties which are developed. One example is the coating of iron oxide nanoparticle with dextran,^135^ which are able MRI-contrast agents and known targets in molecular imaging agents. Moreover, iron oxides nanoparticles which include carboxyl acid at their surface are used for conjugation of proteins and antibodies.^136^

Iron oxide nanoparticles are easily functionalized with a variety of hydrophobic and hydrophilic coating agents such as poly(ethylene glycol)(PEG),^137^ fatty acids, and dextran.^135^ Moreover, some studies have shown that IONPs were functionalized with drugs and fluorescent dye molecules (e.g., BODIPY). Therefore, combination of fluorescent dye with iron oxide nanoparticles conclude to the magnetic-fluorescent nanostructures which have promising medical applications, drug delivery, and imaging.^60,62,138,139^

## 5 Multifunctional and Multimodality Nanoparticles-Based Systems for All-in-One Optical and Nuclear Imaging Applications

**Figure 14:**
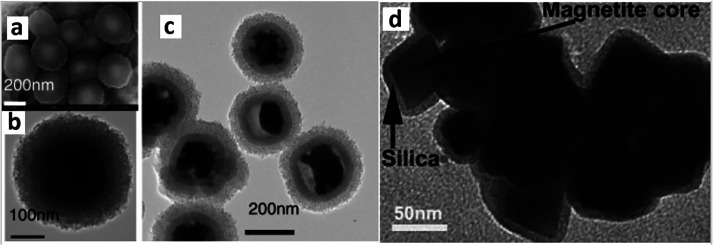
Various magnetic nanoparticles coated with silica shells: Backscattered electron images (a) and TEM images (b) of Fe_3_O_4_-core/SiO_2_-mesoporous-shell magnetic nanoparticles. TEM image (c) of Fe_3_O_4_-core/SiO_2_-mesoporous-shell magnetic nanoparticles. TEM image (d) of Fe_3_O_4_-core/SiO_2_-shell magnetic nanoparticles. Reproduced with permission under a Creative Commons CC-BY license from ref (223). Copyright 2019 The Author.

Core–shell nanoparticles are made up of a core material, such as gold or aluminum, surrounded by a monolayer of material, which can be further functionalized for drug delivery by attaching groups that increase stability and tumor targeting (Figure 14).

The use of a magnetic materials such as iron, nickel, and cobalt as the core material has shown significantly enhanced drug delivery with the aid of an external magnetic field.^140^ This experimental cancer treatment, named magnetic hyperthermia, uses magnetic nanoparticles, which have been shown to damage and kill cancer cells.^141^ The heating method is also particularly useful in protecting healthy tissue, as healthy cells are destroyed at a higher temperature, and so by heating the magnetic nanoparticles to a certain temperature, cancer cells can be destroyed while simultaneously protecting the healthy tissue.^142^ This occurs due to an alternating magnetic field, causing the magnetic nanoparticles to heat, which in turn destroys cancer cells due to their low heat tolerance.

The combination of multiple molecular imaging techniques can also offer synergistic advantages over any modality alone and can be an essential tool in state-of-the-art imaging research as well as standard practice in the clinic.^143^ One of the examples of multimodal imaging are the simultaneous PET-MRI technique. This new approach for functional and morphological imaging was first described by Judenhofer et al.^144^ The synergistic combination of PET and MRI holds promise for the successful next generation of dual-modality scanners in medical imaging. These instruments will provide us with accurate diagnoses thanks to the sensitive and quantifiable signal of PET and the high soft-tissue resolution of MRI.

The standard dual-modal PET-MRI imaging agent was based on a PET isotope and gadolinium.^145^ The second generation of dual (multi)modal contrast agents are synthesized using MNPs, having a proven record of biocompatibility and a track record of extensive use in the clinic as MRI contrast agents.^146,147^

**Figure 15:**
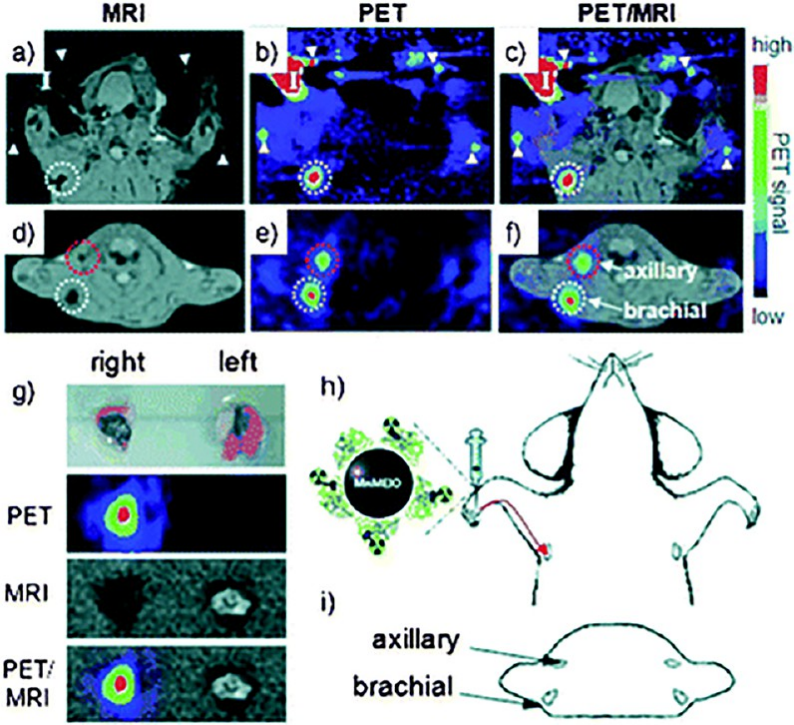
(a–f) PET/MR images of SLNs in a rat at 1 h post injection of ^124^I-SA-MnMEIO into the right forepaw (I = nanoprobe injection site). Coronal (a) MR and (b) PET images in which a brachial LN (white circle) is detected. (c) The position of the brachial LN is well-matched in a PET/MR fusion image. Four small pipet tips containing Na^124^I solution are used as a fiducial marker (white arrowheads) for the concordant alignment in PET/MR images. In the transverse images, axillary (red circle) and brachial LNs (white circle) are detected in the (d) MR and (e) PET images, and images of each node are nicely overlapped in the corresponding PET/MR fusion image (f). (g) The explanted brachial LN also shows consistent results with in vivo images by PET and MR. Only the LN from the right-hand side of the rat containing ^124^I-SA-MnMEIO shows strong PET and dark MR images. The schematics of the rat in the (h) coronal and (i) transverse directions show the locations of the LNs. Reproduced with permission from ref (143). Copyright 2008 John Wiley and Sons.^143^

There are a few early examples of dual (multi)modality described in the literature over the past 10–15 years, which pioneered the use of hybrid nanomaterials for PET/MRI or PET/MRI/NIRF (near-infrared fluorescence). For example, a dual-modal PET/NIRF fluorescent nanotag for long-term immune cell tracking reported by Aras et al.^148^ or the preparation of serum albumin modified MnFe_2_O_4_ nanoparticles conjugated with ^124^I in an early study reported by Choi et al.^143^ (Figure 15). Interestingly, a dual-modal PET/NIRF nanoparticle-based imaging probe consisting of near infrared fluorescent (NIRF) silica nanoparticles containing the silane-appended near-infrared fluorophore (CF-MPTMS)) then radiolabeled by entrapping of the (oxophilic) ^89^Zr-oxalate was utilized for the cell tracking in a mouse model of carcinomatosis. The authors state that such multimodal probe is clinically translatable and the resulting PET/NIRF nanotag-based could assist the direct immune cell labeling approach and act as a synthetic platform that enables whole- body cell tracking over 1 week. Such nanoparticles-based multimodal cell-tracking systems can act as true theranostics and are expected to lead to the next generation of theranostics for future clinical applications.^148^ Also at the start of this field, Lee et al. described amino modified MNPs conjugated to cyclic RGD peptides known to target integrin αvβ3 targeting and simultaneously feature the macrocyclic 1,4,7,10-tetraazacyclododecane-*N*,*N*′,*N*″,*N*′″-tetraacetic acid (DOTA) chelators. This hybrid was designed for PET imaging and labeled with ^64^Cu.^77^ Jarrett et al. further developed in early approaches the ^64^Cu radiolabeling of dextran-based and sulfate-coated superparamagnetic iron oxide nanoparticles.^149^

Earlier studies, by Devaraj et al.,^78^ already reported the synthesis and *in vivo* characterization of ^18^F modified trimodal MNPs (^18^F-CLIO). This particle consisted of cross-linked dextran held together in a core–shell formation by a superparamagnetic iron oxide core and functionalized with the radionuclide ^18^F in high yields via click chemistry. Serum albumin MNPs, dually labeled with ^64^Cu-DOTA and Cy 5.5, were synthesized by Xie et al.^150^ (as a trimodality imaging agent for PET/NIRF/MRI. Glaus et al.^151^ reported synthesis of a probe consisting of a superparamagnetic iron oxide (SPIO) core coated with PEGylated phospholipids. The chelator 1,4,7,10-tetraazacyclo-dodecane-1,4,7,10-tetraacetic acid (DOTA) was conjugated to PEG termini to allow labeling with positron-emitting ^64^Cu. The radiolabeling of MNPs and anchoring of fluorescence dyes has also been included for *in vitro* characterization purposes and common dyes have been included through chemical conjugation. The isotope or dye might be bound relatively weakly to the surface of the MNPs, which might result in a lack of stability over time).^152^

**Figure 16:**
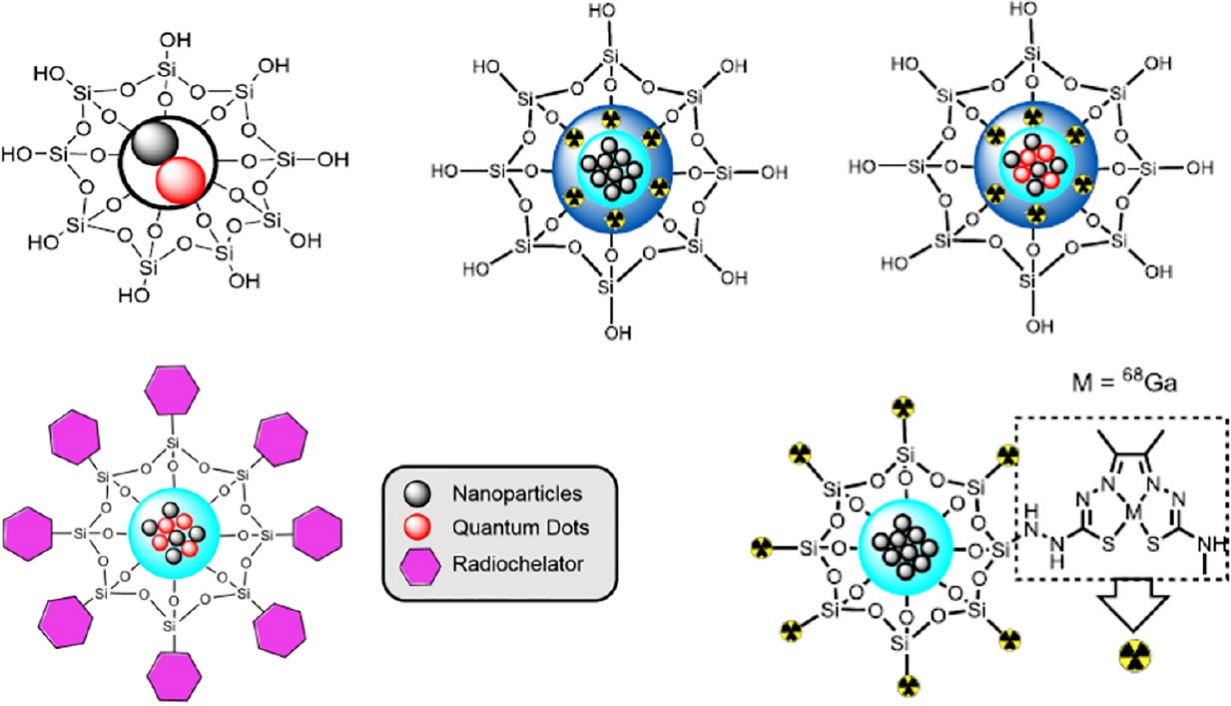
General overview for silica-coated nanoparticles functionalized with fluorescent quantum dots and a chelator from the Lledos, Calatayud, and Pascu state-of-the-art:^84^ Cd_0.1_Zn_0.9_Se QDs modified silica-coated magnetic IONPs; Fe_3_O_4_@SiO_2_@^68^Ga@SiO_2_ (RCY 70%) and Fe_3_O_4_/Cd_0.1_Zn_0.9_Se@SiO_2_@^68^Ga@SiO_2_ (66%), Fe_3_O_4_@SiO_2_@Zn(ATSM/A)@^68^Ga (RCY > 99%).

Novel scalable and postsynthetic surface modification protocols to attach ^64^Cu and ^68^Ga radioisotopes to fluorogenic composite materials incorporating nanoceramics have been reported, building on the evidence in the field that silica materials with magnetic cores have attracted attention in many different areas of research in the past few decades due to the broad range of potential applications they can offer (Figure 16). Covalent and noncovalent synthetic procedures have been designed to obtain magnetic and luminescent biocompatible core–shell siloxane nanoparticles. The potential of such nanocomposites to act as cancer imaging agents in PC-3 cells has been investigated via confocal fluorescence microscopy, UV–vis, FLIM, TCSPC, and MTT assays, as well as DLS, TEM, and EDX.^84^

The encapsulation of nanoparticles within silica nanodimensional layers (or “ensilication”, giving rise to core–shell nanoparticles such as nanoceramics) provides a protective layer that reduces oxygen molecule penetration in both air and aqueous media. This has been highlighted in a range of reports, either focusing on silica alone or with relevance to multimodality imaging as previously mentioned.^153−155^ The decoration of silica nanoparticles with thiols further provided a kinetically stable environment for the immobilization of ^64^Cu. This opens the possibility for incorporating other ligands such as targeting peptides or antibodies in a later stage, as the stability of the sulfur-SNP was deemed to be unaffected by coating with PEG after radiolabeling. Although questions remain over the most effective ways to incorporate the radionuclide into the silica shell, for longer-lived radioisotopes, this presents a smaller technical impediment in practical terms, and the authors suggest that further functionalization should also not affect ^64^Cu stability within these NPs.^153^

Silica is resistant to swelling, which means that the size of the silica particles remains unchanged in a wide range of solvents. The highly oxyphilic nature of the long-lived radioisotope ^89^Zr enables the formation of highly kinetically stable silica-based radio-nanoparticles. Interestingly, these hybrids showed a high *in vivo* integrity, even if these mesoporous silica nanoparticles were assembled and labeled in a chelator-free way with zirconium-89.^154^ The authors raise the necessity of this approach in light of the need to address the long-term *in vivo* integrity for NPs that are intended for nanotheranostics as well as labeled with long-term radioisotopes, and the need to align seamlessly the biodistribution patterns between nanoparticles and radioisotopes behaviors *in vitro*/*in vivo*.

We^84^ and others^132^ have investigated alternative functionalization methods for silica-based tagging with oxyphilic radionuclides and showed the high intrinsic kinetic stability of such constructs. These could complement the traditional chelator-based radiolabeled nanoparticle design for diagnostic techniques taken alone or in multimodality (combining PET/SECT/MRI and optical probes as reported) and/or in radio-nanotheranostic mode. Other advantages of chelator-free methods include the relatively easy incorporation of the tracer and simultaneous functionalization of the nanoparticle surface (being silica-based or carbonaceous layered-based, such as in graphene oxides^156^) providing intrinsic hydrophilicity and allowing surface attachment by covalent binding of many biomolecules for a wide range of applications.^157−159^

Therefore, the development of new imaging tools and scanning techniques requires a new class of imaging probes.^160^

While there has been increasing interest in the development of dual (multi)-modality PET-MRI agents, especially those centered on radio-nanomedicines, this field has expanded even further into the design and preclinical investigations of trimodality probes.^161−163^

## Conclusions

We surveyed and outlined herein the diversity in multimodality function of nanoceramic and related materials, viewed from an applied bio- and nanomaterials chemistry perspective. We highlighted a selection of the new developments in synthesis, radiolabeling, and microscopy investigations as well as some of the current preclinical applications in molecular imaging. We intended to provide an accessible overview of the state-of-the-art and to deliver insights related to multimodal imaging in the context of nanomedicine and radio-nanomedicines, which use synthetic scaffolds such as nanoceramics, which are inorganic oxide-based nanoparticles with ca. 100 nm diameter. The focus is on medical imaging applications (PET/MR and multimodal aspects linking *in vitro* and *in vivo* imaging aspects), from an inorganic and biomaterials chemist’s viewpoint. The primary advantage of nanoparticles, which are the mainstay of nanomedicine, is their ability to deliver multifunctionality. We determined that such an overview would be timely because the total global market of nanomedicine is rapidly growing: the 2022 estimation by some authors projected this growth to reach USD 293.1 billion.^222^ Some of the major breakthroughs and challenges toward the simultaneous incorporation of imaging agents within accessible nanoparticulate materials and the generation of highly kinetically stable nanoparticles as radio-nanomaterials with potential to act as tracers with (pre)clinical theranostic applications are a matter of lively investigations^223−225^ and were included hereby.

Synthetic nanoplatforms show incredible functional diversity, which facilitates their ability to support a variety of biomedical imaging modalities relevant to clinical practice, including optical imaging, computed tomography (CT), magnetic resonance imaging (MRI), single photon emission computed tomography (SPECT), and positron emission tomography (PET).

These diagnostic methods have a well-known range of diversity of advantages and disadvantages, which influences the choice for clinical practice: for example (taken in order of patience accessibility), overall, CT, MRI, and PET have high tissue penetration depths which is combined with spatial resolutions only within the millimeter range. In contrast, optical imaging (fluorescence-emission based, on a range of wavelengths) has high spatial resolution at the subcellular scale but is complemented by a penetration depth limited to only several centimeters (however, advancements in the architecture and testing of new, synthetic, NIR emitting dyes are rapidly addressing these limitations too).

The goal has been, over several decades, to develop synthetic scaffolds that would draw from the advantages of each of these methods and address their disadvantages. Multimodal imaging has generated considerable academic and clinical research and development as well as commercial interest because of the opportunity to use complementary information from different imaging modalities to enhance the accuracy of diagnosis or disease progression for the patent benefit.

A single nanoparticle can incorporate numerous contrast agent units or imaging tracers and encapsulate and/or conjugate to different imaging tags, enabling multimodality diagnostic methods. These arrangements have demonstrated significant improvements in signal-to-noise ratios over molecular imaging techniques such as PET diagnostic imaging with nanomaterials versus molecular species used as radiotracers. Our emphasis was deliberately focused on a class of endogenous biocompatible nanomaterials that are placed at the forefront of some of the primary preclinical developments, such as core–shell materials and nanoceramics, and we wished to compare their potential as nanomedicines with the more rapid advancement liposome-based constructs as theranostics, which have driven the evolution of diagnostic radio-nanomedicines over the past decade.

It is remarkable that new advances in nanotechnology have the potential to facilitate earlier disease detection, increase diagnostic accuracy, and personalize treatments, particularly for noncommunicable diseases (NCDs) such as cancer, yet the number of theranostic constructs based on core–shell nanomaterials (especially those silica-coated, denoted nanoceramics in this report) in clinical trials remains extremely small.

In conclusion, the multifunctional potential synthetic nanoplatforms are a major advantage rendering them useful for nanomedicine design, which can be tailored to support a range of biomedical imaging modalities relevant to clinical practice. There are aspirations toward theranostic applications and addressing the unmet chemical needs in tackling noncommunicable diseases, such as cancer; however, further (pre)clinical studies are necessary before realizing the full potential for patient benefit can be reached. The nanomaterials diversity, structural as well as functional, may be viewed as an advantage, as well as a “poisoned chalice”: a single nanoparticle has the potential to incorporate (single, or in combination) a myriad of contrast agent units and/or imaging tracers, encapsulate, and/or incorporate different combinations of imaging markers, thereby providing the means for multimodal diagnostic approaches and so forth. Therefore, in this review, we focused on some key findings of the simultaneous incorporation of nanoparticulate materials and imaging agents into highly kinetically stable radioactive nanomaterials as potential tracers with (pre)clinical potential. We therefore summarized the functional diversity and new developments in synthesis, radiolabeling, and microscopy studies, with a focus on preclinical applications of molecular imaging aiming to raise the interest in the field of multimodality imaging and tumor nanodiagnostics while remaining grounded into the practical aspects of the biomedical and medicinal materials chemistry aspects of nanotheranostics design.

We remain in awe of the rapidity with which the field of nanomedicine and radio-nanomedicines advanced over the past decade, and it is certain that its further advancements hold great potential for early diagnosis and personalized treatment, especially for hard-to-treat cancers. However, molecular imaging still faces challenges in finding a single modality that can provide all of the essential information required. The combination of multiple reporting probes, such as PET/MR/optical and/or SPECT/MR/optical models, which, combined with (radio) therapeutics delivery in the “all-in-one” approach, could address these challenges and improve the accuracy of molecular imaging for diagnostic as well as therapeutics.

It remains the case that clinical studies involving nanoparticulate materials have shown modest advancements over the past decade, but it is expected that these technologies will continue to improve, particularly for liposome-based nanomedicines, which have recently shown promise as adjuvants for vaccine therapies. The wider applicability of these technologies could be improved by attaching well-understood small molecular tags as “ligands” to the nanoparticle surfaces to increase active targeting of the tumor. However, toxicity of all nanoparticulate constructs remains an issue, particularly in interactions with cell membranes, and surface modifications need to be carefully considered through judicious synthetic and medicinal chemistry approaches at the design stage, including to minimize the effects of surface end groups. It is also necessary to systematically test each nanoparticle treatment group to check for batch-to-batch reproducibility and toxicity issues. Overall, the potential of nanomedicine and radio-nanomedicines is vast, and continued research and development in this field will be crucial in improving the accuracy and efficacy of cancer diagnosis and treatment.
